# Mitochondrial Effects of Common Cardiovascular Medications: The Good, the Bad and the Mixed

**DOI:** 10.3390/ijms232113653

**Published:** 2022-11-07

**Authors:** Alina M. Bețiu, Lavinia Noveanu, Iasmina M. Hâncu, Ana Lascu, Lucian Petrescu, Christoph Maack, Eskil Elmér, Danina M. Muntean

**Affiliations:** 1 Doctoral School Medicine-Pharmacy, “Victor Babeș” University of Medicine and Pharmacy from Timișoara, Eftimie Murgu Sq. No. 2, 300041 Timișoara, Romania; 2 Center for Translational Research and Systems Medicine, “Victor Babeș” University of Medicine and Pharmacy from Timișoara, Eftimie Murgu Sq. No. 2, 300041 Timișoara, Romania; 3 Department of Functional Sciences—Pathophysiology, “Victor Babeș” University of Medicine and Pharmacy from Timișoara, Eftimie Murgu Sq. No. 2, 300041 Timișoara, Romania; 4 Comprehensive Heart Failure Center (CHFC), University Clinic Würzburg, 97078 Würzburg, Germany; 5 Department of Internal Medicine 1, University Clinic Würzburg, 97078 Würzburg, Germany; 6 Mitochondrial Medicine, Department of Clinical Sciences Lund, Faculty of Medicine, Lund University, BMC A13, 221 84 Lund, Sweden; 7 Abliva AB, Medicon Village, 223 81 Lund, Sweden

**Keywords:** cardiovascular drugs, drug toxicity, mitochondria function and morphology, adverse effects, lactic acidosis, drug intoxication, drug interaction

## Abstract

Mitochondria are central organelles in the homeostasis of the cardiovascular system via the integration of several physiological processes, such as ATP generation via oxidative phosphorylation, synthesis/exchange of metabolites, calcium sequestration, reactive oxygen species (ROS) production/buffering and control of cellular survival/death. Mitochondrial impairment has been widely recognized as a central pathomechanism of almost all cardiovascular diseases, rendering these organelles important therapeutic targets. Mitochondrial dysfunction has been reported to occur in the setting of drug-induced toxicity in several tissues and organs, including the heart. Members of the drug classes currently used in the therapeutics of cardiovascular pathologies have been reported to both support and undermine mitochondrial function. For the latter case, mitochondrial toxicity is the consequence of drug interference (direct or off-target effects) with mitochondrial respiration/energy conversion, DNA replication, ROS production and detoxification, cell death signaling and mitochondrial dynamics. The present narrative review aims to summarize the beneficial and deleterious mitochondrial effects of common cardiovascular medications as described in various experimental models and identify those for which evidence for both types of effects is available in the literature.

## 1. Introduction

The heart is the most energy demanding organ of the body with the daily consumption of ATP surpassing the cardiac weight by 5–10 fold. Since the heart derives over 95% of the energy from mitochondrial oxidative phosphorylation, it is not surprising that mitochondria occupy approximately one third of the cardiomyocyte volume [1]. Besides their pivotal role in ATP production, mitochondria have been increasingly recognized as the organelles central to other key processes, such as metabolic control, signal transduction, and cell death [2,3]. Alterations in both mitochondrial function and dynamics have been systematically reported to occur in relation to development and/or evolution of the vast majority of non-communicable diseases [4,5], in particular the cardiovascular disorders [6,7,8,9,10]. This interaction is common, especially in older patients with multiple comorbidities, each of them being treated with several drug classes [11]. The drugs have dose-dependent therapeutic and toxic effects and directly or indirectly modulate cardiac mitochondrial function [12]. The pathomechanisms underlying the side- or off-target effects of cardiac drugs are partially elucidated, but accumulating evidence suggests that mitochondrial impairment plays an important role [13]. Moreover, polypharmacy in elderly and the large variability in individual responses can result in unpredictable drug interactions and potentiation of the side- and/or off-target effects of each drug, thus increasing the propensity for mitochondrial toxicity [14,15]. The degree of toxicity described in the literature varies and depends on the type of drug and the experimental conditions under which investigations have been carried out [12]. Frequently, one drug impairs more than a single aspect of mitochondrial function [16,17]. The electrochemical properties and permeability of the pharmaceutical compounds, and their specific chemical motifs, have been additionally linked with mitochondrial toxicity [18,19,20,21,22].

**Figure 1:**
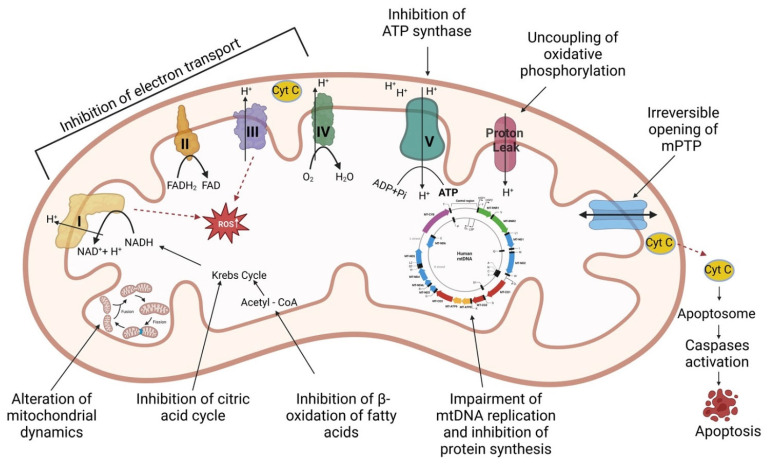
Overview of pathomechanisms of drug-induced mitochondrial toxicity. NAD: Nicotinamide adenine dinucleotide; FAD: flavin adenine dinucleotide; Cyt C: Cytochrome C; ATP: Adenosine triphosphate; mPTP: Mitochondrial permeability transition pore; ROS: Reactive oxygen species. Figure created with BioRender.com.

Drugs can induce mitochondrial toxicity through one or more pathomechanisms, such as inhibition of electron transport system (ETS) protein complexes (including ATP synthase), uncoupling of electron transport from ATP synthesis, irreversible opening of the mitochondrial permeability transition pore (mPTP), inhibition of β-oxidation of fatty acids, inhibition of the citric acid cycle, impairment of either mtDNA replication or mtDNA-encoded protein synthesis, increased oxidative stress, alterations in mitochondrial dynamics [18,20,23] and activation of various mitochondrial-dependent and -independent cell death pathways [24]. An overview of the above-mentioned mechanisms is presented in Figure 1. Moreover, damaged mitochondria trigger inflammatory/immune responses that further contribute to the unfavorable evolution of chronic diseases [25]. In order to repair mitochondrial damage, various mechanisms can be activated, such as mitophagy, fission/fusion, antioxidant defense, and mitochondrial biogenesis associated with transcription, translation, and proteostatic signaling programs, which will further restore the organelles’ homeostasis [1,19].

The most susceptible organs to drug-induced mitochondrial toxicity are the heart, the liver and kidneys. The former is due to its high energy demand and the latter organs are due to their exposure to increased drug concentrations [16,18]. The current narrative review focuses on the mitochondrial effects of specific drugs groups used in the therapeutics of major cardiovascular diseases (hypertension, coronary heart disease, atrial and ventricular arrhythmias, heart failure), based on the data available from experimental and clinical studies.

## 2. Mitochondrial Effects of the Main Classes of Drugs Used in Cardiovascular Diseases

Several classes of pharmaceutical compounds are available to address the complex pathophysiological mechanisms of cardiac diseases, which are largely aimed at both antagonizing the neuroendocrine activation and supporting myocardial contractility and metabolism [26,27]. These drugs have various dose- and model-dependent effects on mitochondrial function/structure, which are systematized herein as beneficial (Table 1), deleterious (Table 2) or mixed (Table 3) effects.

The complex mitochondrial effects of specific cardiovascular drugs in various experimental models are detailed in the following subsections.

### 2.1. Sympathomimetics

#### Isoprotenerol

Isoproterenol is a sympathomimetic agent, which has positive chronotropic and inotropic effects, but when administered in high doses, it can lead to oxidative stress, causing irreversible damage to the membrane, which results in the development of necrosis [221]. In rat heart mitochondria isoprotenerol impaired the functional state of the mitochondria by decreasing the respiratory control index (an indicator of the effectiveness of mitochondrial oxidative phosphorylation), the levels of the main subunits of the respiratory chain complexes and the activity of complexes I, II, IV and that of the ATP synthase [106]. Additionally, in rat heart mitochondria isoprotenerol decreased the concentration of cardiolipin, which plays an important role in the regulation of membrane integrity, reduced the Ca^2+^ retention capacity, thus stimulating the opening of the mPTP which led to an increased rate of mitochondrial swelling [107]. In addition, isoprotenerol-treated rat heart mitochondria showed a significant increase in the levels of lipid peroxidation, calcium ions and a significant reduction in the levels of glutathione peroxidase, decreased glutathione, glutathione-S-transferase, isocitrate, malate, α-ketoglutarate, and succinate dehydrogenases [108,109,110]. In cardiomyoblasts, isoprotenerol induced antioxidant depletion, increased expression of inflammatory markers, DNA damage and apoptotic signaling through upregulating expression of Bax, cytochrome c, Fas, caspase-3, caspase-8, and caspase-9 and downregulating expression of Bcl-2 and Bcl-xL [111,112,113]. In mice, isoprotenerol treatment caused cardiac hypertrophy, reduced protein sulfhydryl content, impaired superoxide dismutase activity and catalase activity, and increased H_2_O_2_ production [114]. Isolated cardiac mitochondria from mice treated with isoproterenol showed a decreased mitochondrial superoxide dismutase activity, and higher mitochondrial Ca^2+^-induced swelling secondary to mPTP opening [114]. In cardiomyocyte mitochondria from rats with experimental chronic heart failure, isoprotenerol induced uncoupling of mitochondrial respiration and decreased ATP production [115].

### 2.2. Antiarrhytmics

#### 2.2.1. Class I (Na-Channel Blockers)

- *Quinidine*

Quinidine is a blocker of the fast sodium channel, classified as a class Ia antiarrhythmic drug that is still used in cardiology for the treatment of ventricular arrhythmias in patients with channellopathies, in particular, the Brugada’s syndrome, early repolarization syndrome and short QT syndrome [222]. It has been reported in the literature that in rat heart mitochondria quinidine slowed down electron transfer activities, uncoupled oxidative phosphorylation and reduced mitochondrial creatine phosphate kinase activity [116]. Concomitantly, the mitochondrial membrane showed a loss of semi-permeability in the presence of quinidine, which was evidentiated by an increase in creatine content [116]. Recently, it has been reported that quinidine partially blocked mitochondrial voltage-dependent anion channel isolated from rat brain [116]. In the presence of quinidine in heart mitochondria, the production of total adenine nucleotides (especially ATP) was shown to decrease to 65% of the normal levels, and protein synthesis was moderately inhibited [117]. In rat renal cells, quinidine reduced respiratory control index, the ADP/O ratio and the oxygen consumption rate [117].

- *Lidocaine*

Lidocaine is a commonly used local anesthetic agent, classified as a class Ib antiarrhythmic drug [223]. In cardiology it is used in the management of acute ventricular tachy-dysrhythmias [224]. Mitochondria are one of the critical targets of lidocaine [225]. It has been reported that lidocaine suppressed the mitochondrial ETS in neuronal SH-SY5Y cells in a dose- and time-dependent manner, and thus attenuated mitochondrial membrane potential, increased reactive oxygen species (ROS) production, and activated caspase-9- and caspase-3/7-mediated apoptosis and necrosis [152]. In human neutrophils lidocaine was shown to suppress their function by reducing the oxidative burst and phagocytosis activity, to inhibit ATP synthesis, to reduce mitochondrial membrane potential and to induce mitochondrial structural changes and apoptosis. This, in contrast, to ropivacaine and bupivacaine, that displayed no effect on neutrophil and mitochondrial functions [153]. In a recent study, lidocaine was shown to alleviate mitochondrial impairment caused by isoflurane, by decreasing the mitochondrial structure damage and the decline in mitochondrial membrane potential [154]. It has also been shown to be successful in reversing isoflurane-induced mitochondrial electron transfer chain dysfunction, as well as in inhibiting the apoptotic activities induced by isoflurane in H4 cells and Fischer 344 rats [154].

- *Phenytoin*

Phenytoin is a sodium channel blocker, classified as a class Ib antiarrhythmic drug, widely used as an anti-seizures drug [226]. In the heart, phenytoin has been reported to inhibit the ectopic rhythm of both the atrium and ventricle and to determine a faster conduction rate of the atrioventricular node in order to decrease myocardial autonomy [226]. In rat hepatocytes phenytoin was shown to increase ROS formation, decrease intracellular reduced glutathione, elevate cellular oxidized glutathione, and amplify lipid peroxidation and mitochondrial impairment [155]. It has been reported in a murine hepatic microsomal system that phenytoin metabolites affected mitochondrial function by reducing state-3 respiration, the respiratory control rate, ATP synthesis, and the membrane potential, by increasing state-4 respiration and also by damaging Ca^2+^ uptake and release, and by inhibiting Ca^2+^ induced swelling [156]. In an animal model of epilepsy, intraperitoneal injection of phenytoin increased superoxide dismutase activity, reduced cerebral malondialdehyde, a biomarker of oxidative stress, and decreased monoamine oxidase A + B activity [157].

- *Propafenone*

Propafenone is class Ic antiarrhythmic agent that is commonly used for treatment of atrial fibrillation in patients with no structural heart disease [227]. In esophageal squamous cell carcinoma, propafenone elicited mitochondrial dysfunction as shown by a reduced mitochondrial membrane potential and decreased expression of Bcl-xL and Bcl-2 and thus being able to suppress cancer cells proliferation in a dose-dependent manner [118].

#### 2.2.2. Class II (β-Blockers)

- *Carvedilol*

Carvedilol is a non-selective β-blocker and an α-blocker that also has antioxidant properties, commonly used for the treatment of hypertension, heart failure or chronic stable angina [228]. Carvedilol antioxidant effects were demonstrated in several studies where it has been shown to inhibit lipid peroxidation in swine ventricular membranes, rat brain homogenates, human LDL, bovine and human endothelial cells, as well as in sonicated phosphatydilcholine liposomes [158]. Both carvedilol and its metabolite BM-910228, at the concentrations at which their antioxidant activity is effective, do not affect mitochondrial function [229]. With regards to mitochondrial oxidative phosphorylation, carvedilol induces a “mild uncoupling” effect [159], a fact that may contribute to mitochondrial protection since small decreases in mitochondrial membrane potential may lower mitochondrial ROS formation and may prevent Ca^2+^ overload in pathological situations, like in ischaemia/reperfusion of the myocardium, since the driving force (membrane potential) for Ca^2+^ uptake is decreased [160]. The protective effect of carvedilol has also been reported against doxorubicin toxicity by inhibiting complex I and by scavenging ROS and, thus, preventing oxidative damage and of the occurrence of mitochondrial permeability transition [158]. In isolated heart mitochondria, carvedilol inhibits mitochondrial permeability transition and protects mitochondria against oxidative stress induced by the xanthine oxidase/hypoxanthine pro-oxidant system [160]. In rat C6 glioma cells, carvedilol induced severe mitochondria damage such as mitochondrial swelling, crista damage and formation of myelin figures inside the mitochondria [161]. In an animal model of type I diabetes, carvedilol increased the level of antioxidant enzymes, thereby contributing to the maintenance of cell redox balance during hyperglycaemia [230]. However, it has to be mentioned that carvedilol concentrations (10–20 μM) used in several studies are much higher than compared to the plasma level of carvedilol reported in patients (24–262 μg/L, corresponding to 0.1–0.6 μM) [231].

- *Nebivolol*

Nebivolol is a third-generation β-blocker with vasodilator function that is widely used for the treatment of hypertension in association with other clinical situations such as angina, heart failure and arrhythmia [232]. In streptozotocin-treated diabetic rats, nebivolol exhibits antioxidant activity via direct free radical scavenging and inhibition of NADPH oxidase activity [162]. In cancer cells, nebivolol was also reported to inhibit complex I and ATP synthase activities and to arrest angiogenesis in order to restrict colon and breast tumor growth [163]. A recent study demonstrated that nebivolol suppressed oral squamous cell carcinoma growth via endoplasmic reticulum stress and mitochondrial dysfunction [164]. Mitochondrial dysfunction was indicated by the significant reduction in the oxygen consumption rate in the oral squamous cell carcinoma cells and more precisely, by the significant decrease in basal respiration, ATP production, and maximal respiration in the nebivolol-treated groups when compared with the control groups [164]. Additionally, nebivolol induced mitochondrial morphology changes and reduced the activity of complex I, which can impair the electron transport chain and result in mitochondrial dysfunction and increased ROS production [164]. The protein expression of OXPHOS complex subunits and the mitochondrial mass were not affected by nebivolol [164].

- *Metoprolol*

Metoprolol is a selective β_1_-adrenoceptor antagonist used for the management of heart failure, chronic stable angina and tachyarrhythmias [228]. In rat cardiomyocytes, metoprolol did not determine protective effects against rat mitochondrial DNA alterations in cardiotoxicity induced by Adriamycin [165]. Metoprolol was ineffective in reducing lipid peroxidation, even at an elevated concentration in vitro [166,167]. In a rat model of ischemia/reperfusion injury, metoprolol enhanced mitochondrial respiratory control ratios in ischemic and nonischemic myocardium [167]. Contrary, in another study that used a rat model of ischemia/reperfusion injury, metoprolol did not show significant improvement in respiratory control ratio or mitochondrial Ca^2+^ content [168]. In addition, metoprolol did not alleviate mitochondrial function in hypertrophied right ventricles of pulmonary hypertensive rats [233].

- *Atenolol*

Atenolol is a β_1_-selective β-blocker, currently used for treating hypertension, chronic stable angina or cardiac arrhythmias [228]. Seyde et al. demonstrated that atenolol increased ROS levels, decreased mitochondrial succinate dehydrogenase activity and the mitochondrial membrane potential, and induced mitochondrial swelling and cytochrome c release in isolated heart mitochondria [119]. Additionally, after the exposure of cardiomyocytes to atenolol, an increase in caspase-3 activity and a decline in the ATP content was noticed [119]. Atenolol also increased the amount of the extracellular-signal-regulated kinase signaling protein, decreased the membrane fatty acid unsaturation degree of mitochondria, lowered mitochondrial protein oxidative, glycoxidative, and lipoxidative modification and reduced oxidative damage in heart mitochondrial DNA [169,170,171]. Therapeutic plasma concentrations of atenolol are between 200–500 ng/mL [234], while the concentrations used in these studies were between 2.5–20 μg/mL.

- *Propanolol*

Propanolol is a nonselective β receptor blocker used for the treatment of hypertension, chronic stable angina or cardiac arrhythmias [228]. Studies in the literature reported that propranolol altered the mitochondrial membrane and morphology, induced mitochondrial swelling, cytochrome c release, and activation of caspase cascade and apoptosis cell death [120,121,122,123,124]. Similar results were reported by a recent study performed in isolated rat heart mitochondria, where propranolol was shown to damage mitochondria via the inhibition of complex II of the respiratory chain, increase in ROS formation, collapse of the mitochondrial membrane potential, mitochondrial swelling and cytochrome c release [119]. Moreover, propranolol enhanced caspase-3 activity and decreased ATP levels in rat cardiomyocytes [119]. Of note, propanolol concentrations used in these studies (2.5–20 μg/mL) were much higher than plasma concentrations of patients chronically treated with propranolol (5.3 to 300 ng/mL) [235].

- *Timolol*

Timolol is a non-selective β-adrenergic blocker used in topical administration to reduce intraocular pressure in patients with open-angle glaucoma [236] and in systemic administration for the management of hypertension [237]. In an in vitro comparison with other β-blockers, a direct ROS scavenging action of timolol was reported, thus being possibly useful in preventing oxidative damage [28,29]. In a cell culture study, timolol protected against increased oxidative stress [30]. Moreover, in a female rat model of aging-related altered left ventricular function, timolol had a cardioprotective role by preventing antioxidant system dysfunction, including enhanced lipid peroxidation, decreased ratio of reduced glutathione to oxidized glutathione, and lowered activities of thioredoxin reductase and glucose-6-phosphate dehydrogenase of the heart samples [31]. Cicek et al. demonstrated in diabetic rats that timolol alleviated hyperglycemia-induced cardiac impairment by the inhibition of endoplasmic reticulum stress [238]. Timolol prevented the alterations in mitochondria and nucleus of the cardiomyocytes while it determined a well-controlled redox-state and apoptosis in cardiac tissue [238].

- *Esmolol*

Esmolol is a β1-adrenergic antagonist used for controlling supraventricular tachycardia [239]. In spontaneously hypertensive rats, esmolol reduced left ventricular hypertrophy by improving the morphology and stereology of mitochondria [172]. Yardımcı et al. reported that, when applied in the highest concentration, esmolol induced in MRC-5 human lung fibroblast cells a significant increase in ROS levels and a decrease in the mitochondrial membrane potential, although this decrease was not significant [173]. In the literature, several studies reported protective effects of esmolol against apoptosis generally by decreasing the Bax/Bcl-2 levels [173,174,175] in early sepsis rats with abdominal infection [174] and in rat cerebral cortex following controlled hypotension [175].

#### 2.2.3. Class III (K-Channel Blockers)

- *Amiodarone*

Amiodarone is the most potent class III antiarrhythmic drug, commonly used for the management of both ventricular and supraventricular arrhythmias [240]. Amiodarone mitochondrial toxicity was in the literature primarily reported in murine models where amiodarone determined uncoupling of oxidative phosphorylation at lower concentration, inhibition of the mitochondrial complexes I and II of the electron transport system in higher doses, and also inhibition of the fatty acid β-oxidation [125,126,127,128]. Amiodarone was also reported to decrease the intracellular ATP content both in vivo in a rat model of hepatotoxicity [129] and in vitro in isolated rat liver mitochondria, human hepatocytes [130] and in rat H9c2 cardiomyocytes [131]. A recent study has shown that acute administration of amiodarone induced a concentration-dependent mitochondrial dysfunction in human platelets, peripheral blood mononuclear cells and HepG2 cells by inhibiting both CI- and CII-supported respiration [33]. Additionally, in peripheral blood mononuclear cells, amiodarone determined a severe concentration-dependent ATP depletion [33]. Since the latter study was purported to investigate the drug toxicity, it must be mentioned that amiodarone was applied in concentrations varying between 20 and 400 μM (while plasma level of amiodarone is in the range of ~2μM) [33].

- *Dronedarone*

Dronedarone, a non-iodinated benzofuran derivative of amiodarone, classified as a class III antiarrhythmic drug is used for the treatment of atrial fibrillation and atrial flutter [241]. Dronedarone is known for inducing hepatotoxicity mainly via the inhibition of carnitine palmitoyltransferase I and thus of the mitochondrial fatty acid β-oxidation instead of the mitochondrial respiratory chain [129,130,132]. In rat cardiomyocytes, dronedarone was found to damage mitochondria by dissipating mitochondrial membrane potential, inhibiting mitochondrial complex I, uncoupling the mitochondrial respiratory chain and by decreasing the intracellular ATP content [131]. A study performed in HepG2 cells reported the contribution of DNA damage induced-apoptosis to dronedarone-induced cytotoxicity, with the involvement of the activation of caspase-2 and JNK/p38 signaling pathway [241]. Again, the therapeutic serum concentration for dronedarone is around 0.2 μM, while the concentrations used in the ex vivo experiments that reported mitochondrial toxicity are much higher [130].

- *Ibutilide*

Ibutilide, a potassium channel blocker, classified as a class III antiarrhythmic drug, is commonly used in the treatment of atrial fibrillation [242]. In H_2_O_2_-induced apoptosis of neonatal rat cardiomyocytes, ibutilide was shown to have a protective role via suppression of the endoplasmic reticulum and mitochondrial stress pathways [32]. Ibutilide attenuated oxidative stress and mitochondrial-related apoptosis by significantly increasing the levels of glutathione peroxidase, superoxide dismutase and decreasing the levels of malondialdehyde and by lowering the ratio of Bax/Bcl-2 in H_2_O_2_-induced neonatal rat cardiomyocytes [32].

- *Sotalol*

Sotalol, a non-selective β-adrenergic blocking agent classified as a class III antiarrhythmic agent due to its predominant potassium channel blocking effect is used for the treatment of supraventricular arrhythmias, atrial fibrillation/flutter as well as for the management of ventricular arrhythmias [243]. Recently, in human platelets, it has been demonstrated that sotalol did not elicit mitochondrial dysfunction in acute administration [33].

- *Dofetilide*

Dofetilide, a class III antiarrhythmic drug that selectively blocks potassium channels, was associated with an increased susceptibility to life-threatening ventricular arrhythmias [244]. In a heart failure rat model, dofetilide attenuated isoprotenerol-induced heart failure by correcting the abnormal expression of the calcium handling FK506 binding protein, NADPH oxidase and protein kinase C epsilon signaling pathway [34].

#### 2.2.4. Class IV (Ca-Channel Blockers)

- *Verapamil*

Verapamil is a non-dihydropyridine calcium channel blocker, classified as class IV antiarrhythmic drug used for the management of supraventricular tachycardia, hypertension and angina pectoris [35]. Verapamil has been shown to inhibit lipid peroxidation, increase antioxidant enzyme activity and to protect against ROS in diabetic nephropathy [36]. Recently, in a rat model of transient global forebrain ischemia/reperfusion, verapamil elicited neuroprotective effects by decreasing mitochondrial damage and apoptosis [35]. Amelioration of mitochondrial function was indicated by the reduction in ROS formation and cytochrome c release, together with the increased ATP concentration, decreased mitochondrial swelling and prevention of mitochondrial membrane potential reduction in the verapamil treated group as compared to the ischemia/reperfusion group [35]. The beneficial role of verapamil in improving the antioxidant capacity of neurons was supported by the increase in all the antioxidants measured (superoxide dismutase, glutathione peroxidase, glutathione, catalase) [35]. In addition, in human neuroblastoma cells, pre-treatment with verapamil was found to offer protection against scopolamine-induced oxidative injury and mitochondrial impairment [245]. Additionally, verapamil is known for being able to inhibit mitochondrial phospholipase activity, which is linked with mitochondrial swelling and changes in Ca^2+^ flux pathways [245]. In Candida albicans, verapamil also had an inhibitory effect on oxidative stress response [246].

- *Diltiazem*

Diltiazem is a non-dihydropyridine calcium channel blocker, classified as a class IV antiarrhythmic drug that is also used as a antihypertensive and anti-anginal medication [247]. In a model of ischemia/reperfusion using rabbit hearts, diltiazem protected mitochondrial integrity and function and thus preserved myocardial high energy phosphates levels [37]. Similar results were also reported by Kavanaugh et al. in the rabbit heart where the authors found that diltiazem provided a protective effect on myocardial high-energy phosphate metabolism during regional ischemia and reperfusion [38]. In ischemic and reperfused rat hearts pretreated with diltiazem, it improved recovery of contractile function and prevented mitochondrial swelling, structural grade alteration, and increase in mitochondrial Ca^2+^ [39]. Additionally, diltiazem was suggested to decrease lipid peroxidation in reperfused isolated rabbit hearts [40]. In rat hepatocytes, diltiazem inhibited hypoxia-reoxygenation induced JNK(1)/SAPK(1) activation and decreased apoptosis by this mechanism [41].

#### 2.2.5. Others

- *Adenosine*

Adenosine is purine nucleoside base, classified as a miscellaneous antiarrhythmic drug outside the Vaughan-Williams classification scheme. It is currently used as a diagnostic agent in myocardial perfusion stress imaging for its vasodilatory effects as well as a therapeutic drug in paroxysmal supraventricular tachycardia [248]. In rat cardiomyocytes, adenosine was shown to prevent oxidant-induced mitochondrial dysfunction by producing nitric oxide [249]. In human microvascular endothelial cells, adenosine was also reported to reverse TNFα-induced deficits in mitochondrial mass and function, as well as the increase in apoptosis, effects that were mediated via the activation of an eNOS-PGC-1α regulatory pathway [250]. The beneficial effect of adenosine on mitochondrial function was also reported in an experimental model of rabbit heart ischemia/reperfusion, where adenosine attenuated the decline of complex I and mitochondrial nitric oxide synthase activities and reduced mitochondrial phospholipid oxidation [176]. Studies in the literature has reported that adenosine can induce apoptosis in tumor cells [177,178,179]. In liver cancer cells, apoptosis was determined by the increased ROS resulting in mitochondrial dysfunction [177]. In HepG2 cells, extracellular adenosine induced apoptosis by reducing Bcl-x(L) expression and increasing Bid expression, and by that disrupting mitochondrial membrane potentials to release cytochrome c from the mitochondria, and then causing activation of caspase-9 and the effector caspase-3, as mediated by A(2a) adenosine receptors [178].

- *Digitalis*

Digitalis glycosides (digoxin, ouabain) are known to inhibit the Na^+^/K^+^-ATP enzyme and have been used mainly for the treatment of heart failure and for the rate control in atrial fibrillation [251,252]. Studies reported that part of the classical digitalis toxicity may be due to altered mitochondrial energetics and redox balance as a result of digitalis elevating cytoplasmic Na^+^, reducing mitochondrial Ca^2+^ accumulation, decreasing the NADH/NAD^+^ redox potential, and enhancing ROS level [180,181]. On the contrary, Campia et al. reported the beneficial effects of digoxin and oubain in cardiomyocytes by enhancing the efficiency of mitochondrial electron transport and ATP synthesis [182]. A recent study performed in human non-small cell lung cancer cells A549 reported that digoxin induced mitochondria-mediated apoptosis by reducing the mitochondrial membrane potential of these cells [183]. Digoxin also promoted apoptosis in a breast cancer cell line by increasing the intracellular Bax/Bcl-2 proportion, resulting in perforation of mitochondrial membrane, and inducing downstream cascaded events linked with apoptosis [184]. The apoptotic effect of ouabain could be associated with mitochondrial dysfunction, since oubain caused mitochondrial redistribution and disruption, ATP depletion, mitochondrial cytochrome c release and activation of caspase 9 in HeLa cell line [185].

### 2.3. Renin-Angiotensin-Aldosterone System (RAAS) Blockers

The renin-angiotensin-aldosterone system (RAAS) plays a key role in cardiovascular hemodynamics by regulating blood pressure and volume homeostasis [253]. The use of RAAS blockers is considered the first-line therapy in patients with hypertension, heart failure, post–myocardial infarction states, and renal disease [254].

#### 2.3.1. Angiotensin-Converting Enzyme Inhibitors (ACEI)

- *Ramipril*

Ramiprilat and losartan were reported to inhibit cardiac uncoupling protein-2 expression following myocardial ischemia reperfusion in rats [186]. In addition, ramipril was found to attenuate lipid peroxidation in an experimental model of rheumatoid arthritis [187].

- *Zofenopril*

In a rabbit model of ischemia/reperfusion, zofenopril elicited a cardioprotective effect by preventing mitochondrial calcium overload, maintaining oxidative phosphorylating capacities, ATP production and membrane integrity and by decreasing oxidative stress [42]. Additionally, zofenoprilat modulated angiotensin I receptor expression through Sirtuin 1 downregulation [255].

- *Perindopril*

In a rat model of isoproterenol-induced cardiomyopathy, perindopril significantly lowered ROS synthesis, elevated the levels of antioxidant enzymes, alleviated mitochondrial disruption while increasing the number of mitochondria, attenuated the mitochondrial respiratory chain dysfunction, and elevated ATP production. Moreover, perindopril reduced myocardial apoptosis by suppressing cytochrome C leakage from mitochondria and caspase-3 activation in the cytosol [43]. In addition, perindopril was shown to totally prevent ischemia-induced alterations of skeletal muscle mitochondrial function and protein expression in rats [256]. In a pig model of ischemia/reperfusion, perindopril elevated calcium retention capacity, but no decline in the level of ROS production was noticed [44].

- *Captopril*

In rat models of adriamycin toxicity, captopril attenuated the dissipation of mitochondrial membrane potential and increased the ATP production, thus improving the mitochondrial function [188,189,190]. In rabbits with experimentally induced hypercholesterolemia treatment with captopril restored mitochondrial oxygen consumption albeit it did not elicit beneficial effects on serum lipid levels [191]. Others indicated that captopril elicited an antioxidant effect [192]. In contrast, captopril treatment did not elicit any protective effect on mitochondrial function as evidenced by the decreased oxidative phosphorylation rate and lowered ATP production in heart and kidney of spontaneously hypertensive rats [257,258]. Accordingly, Kancirová et al. demonstrated that in vitro captopril inhibited the ATP synthase activity, while in vivo it elicited no direct effect on mitochondrial bioenergetics [259].

- *Trandolapril*

Trandolapril treatment induced significant improvement in mitochondrial enzyme activities (I, II and IV) and attenuated oxidative stress by decreasing lipid peroxidation and increasing levels of catalase, reduced glutathione in rat brain [45]. Trandolapril also prevented mitochondrial dysfunction following acute myocardial infarction in rats by alleviating the decrease in the mitochondrial oxygen consumption rate and ATP production, as well as the increase in the mitochondrial thiobarbiturate-reacting substance content [46,47].

- *Lisinopril*

In a rat model of irradiation-induced kidney damage, lisinopril improved mitochondrial metabolism by attenuating the oxidation of mitochondria leading to increased redox ratio [193]. Lisinopril treatment was reported to modulate age-related mitochondrial metabolic parameters by decreasing mitochondrial respiration and H_2_O_2_ levels and by increasing mitochondrial content in Drosophila melanogaster [194]. In addition, lisinopril was suggested to modulate exercise-induced mitochondrial gene expression in human volunteers [260].

- *Enalapril*

In rat cardiomyocytes, enalapril and losartan were found to enhance mitochondrial nitric oxide synthase activity, and thus to modulate mitochondrial respiration and ROS generation [261]. Furthermore, enalapril enhanced superoxide dismutase 2 [56] and glutathione-dependent [48] antioxidant defenses, and enhanced renal content of the mitochondrial ROS modulator uncoupling protein-2, leading to reduced production of hydrogen peroxide [49]. Administration of a non-antihypertensive dose of enalapril attenuated oxidative stress-induced damage (i.e., mtDNA damage, mtDNA4834 deletion, and protein carbonylation), while increasing mitochondrial mass, mitochondrial biogenesis and promoting mitochondrial fusion and autophagy in aged rat hearts [50]. Recently, in a rat model of heart failure, enalapril was reported to attenuate lipid peroxidation, and preserve protein expression of endogenous antioxidants (Manganese superoxide dismutase and catalase) together with electron transport chain complex activity [51]. In addition, enalapril attenuated doxorubicin-induced cardiomyopathy by improving mitochondrial respiratory efficiency and by lowering the free radical production [52].

#### 2.3.2. Angiotensin Receptor Blockers (ARBs)

- *Valsartan*

In pigs with renovascular hypertension, valsartan was reported to efficiently decrease blood pressure and alleviate left ventricular remodeling, while improving myocardial mitochondrial biogenesis and mitophagy [53]. In rats with type 2 diabetes, valsartan increased the mitochondrial respiratory function in liver mitochondria, and thereby ameliorated the pathological progression of hepatic fibrosis [54]. In addition, valsartan was found to improve mitochondrial dysfunction induced by a high-fat diet in the pancreatic islets of mice [262]. In Ren2 rats characterized by elevated endogenous levels of angiotensin II, valsartan treatment attenuated mitochondrial oxidative damage and increased mitochondrial β-oxidation [55].

- *Losartan*

In spontaneously hypertensive rats, losartan alleviated renal mitochondrial dysfunction by reducing oxidative stress as revealed by increased mitochondrial membrane potential, nitric oxide synthase, manganese-superoxide dismutase and cytochrome oxidase activities, as well as by reduced mitochondrial H_2_O_2_ production and enhanced uncoupling protein-2 content [56]. Similar results were reported by the same authors in streptozotocin-induced diabetic rats [263]. Losartan was also shown to protect against both age-related mitochondrial dysfunction and ultrastructural alterations in aged rats [264]. Long-term administration of losartan ameliorated the decrease in mtDNA content but failed to prevent the age-dependent accumulation of liver mtDNA ‘common deletion’ in rats [57]. Recently, it has been reported that losartan improved mitochondrial dysfunction and biogenesis by upregulating SIRT1, PGC1α, UCP1, and mRNA of Tfam, Cd137, Tmem26, Ucp1 expression in obese mice [58].

- *Candesartan*

Recently, candesartan was found to attenuate mitochondrial dysfunction and ROS production, regulate mitochondrial dynamics by suppressing dynamin-related protein 1 activation and induce Rab9-dependent alternative autophagy in order to alleviate oxidized low-density lipoprotein-induced cellular senescence in vascular smooth muscle cells and in apolipoprotein E-deficient mice [59]. In spontaneously hypertensive rats, candesartan alleviated cardiac remodeling by improving mitochondrial structure, function and dynamics as revealed by the ameliorated mitochondrial morphology, increased mitochondrial membrane potential, enhanced NADH and cytochrome c oxidoreductase activities, reduced manganese superoxide dismutase activity and upregulated the expression of Mitofusin2 [60]. Similarly, De Cavanagh et al. previously reported the beneficial effects of candesartan on improving mitochondrial function in rat kidney mitochondria [265]. In addition, candesartan was reported to elicit neuroprotective effects in a rat model of cerebral ischemia by alleviating oxidative damage and mitochondrial enzyme dysfunction of all respiratory complexes [61].

- *Irbesartan*

Recently, irbesartan was reported to inhibit the mitochondrial apoptotic pathway by reducing the expression of the Bax, tBid, active caspase-9 and -3, and therefore to attenuate sleep apnea-induced cardiac apoptosis [62]. In an in vitro model of non-alcoholic fatty liver disease consisting of free fatty acid-treated hepatocytes, irbesartan attenuated lipid deposition and mitochondrial dysfunction by increasing ATP production and the mitochondrial membrane potential, and by lowering ROS production [63]. Moreover, the authors found that irbesartan enhanced autophagy via the PKC/AMPK/ULK1 axis [63].

- *Telmisartan*

Recently, in a mouse model of Parkinsonism, telmisartan was demonstrated to improve mitochondrial functions by upregulating mitochondria-specific genes expression [64]. In addition, in renal glomerular endothelial cells, telmisartan elicited a protective effect against high-glucose-induced injury by ameliorating mitochondrial dysfunction and oxidative stress, as evidenced by the increased mitochondrial membrane potential and the reduced levels of 8-hydroxy-2 deoxyguanosine (8-OHDG) and malondialdehyde [65]. In cultured human coronary artery endothelial cells, telmisartan enhanced mitochondrial function and elicited anti-senescence effects through AMP-activated protein kinase activation [266]. Telmisartan was reported to modulate mitochondrial Ca^2+^ homeostasis, ROS generation, and mitochondrial energy metabolism through targeting transient receptor potential channel, canonical type 3, in spontaneously hypertensive rats [66]. In human vascular smooth muscle cells, telmisartan enhanced ATP synthesis and mitochondrial complex II activity, lowered H_2_O_2_ levels and caspase 3/7 activity, thus reducing cellular apoptosis, as compared to eprosartan, which elicited no effect on these mitochondria-related cellular responses [67].

- *Olmesartan*

Olmesartan ameliorated the impairment on mitochondrial function and oxidative stress by increasing the mitochondrial enzyme activities of aconitase, complex I, and complex II and the activities of total superoxide dismutase and catalase in the hearts of insulin resistant rats during an acute glucose challenge [68]. In addition, olmesartan administration prevented tacrolimus-induced renal damage by reducing oxidative stress and by reversing ultrastructural mitochondrial alterations [267]. In a model of high-fat diet-induced diabetic mice, olmesartan improved ADP-dependent mitochondrial respiration, as well as NAD(P)H oxidase activity and superoxide production [69].

- *Azilsartan*

Azilsartan was reported to attenuate oxidative injury in murine brain endothelial cells by inhibiting lipid peroxidation and ROS production and by improving mitochondrial function as revealed by elevated mitochondrial membrane potential, reduced cytochrome c leakage, preserved ATP production and reduced mitochondrial swelling [70]. In a rat model of cerebral ischemia, azilsartan was able to alleviate mitochondrial enzyme system impairment (complexes I, II and IV) and mitochondrial viability, and in combination with the ubiquitous electron carrier coenzyme Q10, it potently increased mitochondrial respiration as evidenced by enhanced state III/state II ratio [71]. Moreover, azilsartan lowered apoptosis by decreasing caspase 3 expression and mitigated oxidative stress, by decreasing levels of malondialdehyde and nitrite, and by increasing levels of glutathione and superoxide dismutase [71].

#### 2.3.3. Angiotensin Receptor Neprilysin Inhibitor (ARNi): Sacubitril/Valsartan

Sacubitril/valsartan, the first drug from the new class of drugs called ARNi, whose mechanism of action includes angiotensin II receptor blockade and neprilysin inhibition [72], is currently recommended by the 2021 European Society of Cardiology guidelines for the treatment of heart failure [268]. In the setting of pressure overload, both in vivo and in vitro experiments, sacubitril/valsartan was found to improve mitochondrial function and to elicit a higher protective effect than valsartan in attenuating oxidative stress in ventricular myocytes [72]. In dogs with experimental cardiorenal syndrome, sacubitril/valsartan improved mitochondrial state-3 respiration, mitochondrial membrane potential, attenuated mPTP opening, enhanced the maximum rate of ATP production and normalized the enzymatic activities of complex-I and IV of the respiratory chain [73]. Additionally, it lowered the levels of cytosolic cytochrome c and active caspase-3, thereby mitigating apoptosis and normalized the expression of PGC-1α, an important co-transcriptional regulator of mitochondrial biogenesis [73]. Both in H_2_O_2_-exposed cardiomyocytes and in a rat model of cardiorenal syndrome, sacubitril/valsartan was reported to elicit a protective effect against oxidative damage and to improve cardiac function through regulating Mitofusin2-mediated mitochondrial functional integrity [269].

### 2.4. Calcium Channel Blockers-Dihydropyridines

#### Amlodipine

Amlodipine is a calcium channel blocker commonly used as a first-line agent in the treatment of hypertension [270]. In a pig ischemia/reperfusion model, the preservation of mitochondrial function and structure by amlodipine was demonstrated by increased oxygen consumption at state 3, improved calcium retention capacity and reduced ROS production as well as by reduced mitochondrial swelling [44]. Other mitochondrial beneficial effects of amlodipine have been explained by its antioxidant properties: increased activity of the antioxidizing enzymes glutathione peroxidase, catalase and superoxide dismutase and decreased malondialdehyde levels accounting for reduced lipid peroxidation in cholesterol-induced rabbit model of atherosclerosis, a liver and a heart model of ischemia/reperfusion injury in rat [74,75,76,77]. Additionally, amlodipine was recently shown to inhibit apoptosis and to protect mitochondria against oxidative damage in neural stem cell exposed to oxygen glucose deprivation by reducing cellular and mitochondrial calcium influx, activating the PI3K pathway, enhancing expression of mitochondrial biogenesis-related proteins (such as mitofusin) and survival-related protein Bcl-2, and by decreasing expression of apoptosis-related protein Bax, and cytosolic cytochrome c [78].

### 2.5. Antithrombotic Agents

#### 2.5.1. Acetyl-Salicylic Acid

Acetyl salicylic acid has been widely used as an antithrombotic drug for the treatment and prevention of cardiovascular diseases as well as an anti-inflammatory and analgesic medication [133]. Both salicylic acid and acetyl-salicylic acid were demonstrated to inhibit oxidative phosphorylation and ATP synthesis in isolated rat cardiac mitochondria in a dose-dependent manner [133]. In isolated liver and kidney mitochondria, salicylic acid was reported to act as an uncoupler of oxidative phosphorylation as well as an inhibitor of ADP-dependent mitochondrial respiration [133]. Additionally, acetyl-salicylic acid inhibited the respiratory chain ATPase, resulting in decreased ATP production in rat liver mitochondria [134]. In freshly isolated rat kidney mitochondria, Nasser et al. reported that salicylate opened the mitochondrial transition pore and thus, elicited swelling, the collapse of the mitochondrial membrane potential and mitochondrial calcium release [135].

#### 2.5.2. Clopidogrel

Clopidogrel, a P2Y12 inhibitor, has an essential role in antiplatelet therapy and thus in the treatment and secondary prevention of cardiovascular diseases [271]. An in vitro study performed in isolated mice liver mitochondria showed that when applied in very high doses clopidogrel significantly decreased mitochondrial respiratory state 3 and state 4 respiration and prolonged oxygen consumption in State 3, indicating that mitochondrial oxidative phosphorylation was compromised, as compared to the human therapeutic doses of clopidogrel which did not impaired mitochondrial respiration [136]. Clopidogrel cytotoxicity was also reported in primary human hepatocytes and in HepG2 cells via reduced cellular glutathione content by clopidogrel reactive metabolites as well as mitochondrial impairment and ROS accumulation, eventually resulting in apoptosis [137]. Maseneni S et al. noted that in human neutrophil granulocytes and lymphocytes clopidogrel was able to reduce the membrane potential of the inner mitochondrial membrane, enhance the ROS production, induce cytochrome c release and apoptosis [138].

#### 2.5.3. Ticagrelor

Ticagrelor, a P2Y12 receptor antagonist, is recommended as the first-line treatment in patients with acute coronary syndrome at moderate-to-high risk of ischemic events [272]. Recently, an in vitro study performed in insulin-resistant H9c2 cardiomyocytes has demonstrated that ticagrelor alleviated the insulin resistance-induced mitochondrial damage by improving mitochondrial membrane potential, decreasing ROS production, preserving cellular ATP synthesis, reversing the increased resting level of cytosolic free Ca^2+^, as well as mitigating the mitochondrial ultrastructural changes (swelling and loss of crista) [79]. Moreover, the protective effects of ticagrelor were confirmed in a rat model of metabolic syndrome, where ticagrelor augmented the function and ultrastructure of mitochondria, as well [80].

#### 2.5.4. Prasugrel and Ticlopidine

Prasugrel, a newer P2Y12 blocker, is more clinically effective than clopidogrel or ticagrelor, but is also associated with a higher risk of bleeding [273]. Ticlopidine, a first generation thienopyridine, is less used today due to its potentially fatal side effects, including aplastic anemia, neutropenia and thrombotic thrombocytopenic purpura [274]. In human neutrophil granulocytes and lymphocytes, both prasugrel and ticlopidine were proven to be mitochondrial toxic by decreasing the mitochondrial membrane potential, increasing the ROS accumulation, and thus leading to loss of mitochondrial cytochrome c, activation of caspase 9 and apoptosis in a concentration-dependent manner [138].

### 2.6. Oral Anticoagulants

#### 2.6.1. Coumarin Derivatives

Coumarins are vitamin K antagonists, of which warfarin is the most commonly prescribed for treatment or prevention of deep vein thrombosis and pulmonary embolism or for thromboembolism prophylaxis in patients with atrial fibrillation or other cardiac condition, but with a narrow therapeutic window [275]. In the literature warfarine was reported to induce mitochondrial damage in lymphocytes [276] and lower the cellular ATP content of hepatocytes, resulting in impaired viability [139].

#### 2.6.2. Direct Oral Anticoagulants

Direct oral anticoagulants are drugs prescribed for decreasing the risk of stroke and embolism in atrial fibrillation as well as for deep vein thrombosis and pulmonary embolism treatment/prophylaxis. They are classified into 2 main classes: oral direct factor Xa inhibitors, (rivaroxaban, apixaban, edoxaban, and betrixaban) and direct thrombin inhibitors (dabigatran) [277]. It has been shown that rivaroxaban may protect mitochondria by altering expression levels of an array of genes associated with mitochondrial function in angiotensin II-infused KKAy mice, as well as by alleviating angiotensin II-induced decline in cardiac ROS level and ATP production [278]. In rat kidney mitochondria, the reported effects of rivaroxaban were dose-dependent as follows: at low concentrations, the drug induced mitochondrial dysfunction and oxidative stress by decreasing the activity of mitochondrial succinate dehydrogenase and the mitochondrial membrane potential, and increasing ROS production, mitochondrial swelling, and cytochrome c release, while at high concentrations all these effects were prevented [195]. Previous studies performed in proximal tubular cells exposed to advanced glycation end products (131) and in intermittent hypoxia-exposed mice (132) also showed that rivaroxaban was able to reduce ROS generation [196,197]. Additionally, in human abdominal aortic aneurysms, rivaroxaban was reported to improve mitochondrial function associated with modifications in proteins related to mitophagy [279]. Apixaban was proven to exhibit antioxidant properties by decreasing ROS production in an in vitro model of endothelial dysfunction in uremia [81]. In a rat gastric epithelial cell line, dabigatran elicited cytotoxic effects that were mediated via enhanced ROS generation, reduction in the mitochondrial membrane potential, and increased lipid peroxidation [140]. In human alveolar epithelial cells, edoxaban prevented activated clotting factor X induced-mitochondrial impairment by increasing the mitochondrial oxygen consumption during maximal oxidative phosphorylation and thus the mitochondrial ATP generation [82].

### 2.7. Diuretics

#### 2.7.1. Loop Diuretics

Loop diuretics (furosemide, bumetanide, torasemide) are Na-K-2Cl cotransporter inhibitors, of which furosemide is the most commonly prescribed for the treatment of edema, hypertension and renal conditions [280]. In a mouse model of hepatotoxicity, furosemide did not inhibit mitochondrial respiration supported by complex I or II for up to 5 h following dosing and did not reduce mitochondrial or cytosolic glutathione, suggesting that furosemide-induced hepatotoxicity is not induced by mitochondrial dysfunction [281]. On the contrary, the study of Church et al. revealed that furosemide treatment resulted in mitochondrial damage in another mouse model of hepatotoxicity [282]. In isolated rat kidney mitochondria, furosemide inhibited oxidative phosphorylation, specifically at complex II of the respiratory chain [141]. In addition, furosemide was shown to inhibit state 3 (ADP-dependent) respiration of the rat liver, renal cortex, renal medulla mitochondria [142]. In astrocytes following in vitro ischemia, bumetanide alleviated mitochondrial dysfunction and cell death by attenuating reoxygenation-induced mitochondrial Ca^2+^ overload, dissipation of mitochondrial membrane potential and cytochrome c release [83,84].

#### 2.7.2. Antagonists of Aldosterone

Antagonists of aldosterone (spironolactone, eplerenone) are potassium-sparing diuretics generally used in the management of hypertension, heart failure and post-myocardial infarction [283]. In osteoblastic MC3T3-E1 cells, spironolactone was demonstrated to attenuate mitochondrial dysfunction induced by methylglyoxal by improving the mitochondrial membrane potential, ATP synthesis, proliferator-activated receptor gamma coactivator 1α level, and nitric oxide production. Additionally, it decreased methylglyoxal-induced endoplasmic reticulum stress, cardiolipin peroxidation, the generation of ROS and mitochondrial superoxide levels [85]. Similar findings were shown in another study which used both in vivo and in vitro models, where spironolactone regulated the expressions of key genes involved in the oxidative and antioxidative stress systems [284]. Spironolactone was also reported to protect endothelial cells from apoptosis by inhibiting caspase-3 activity, cytochrome c release and PARP cleavage [86]. In an in vivo study, eplerenone prevented aldosterone-induced cardiac mitochondrial alteration by reversing the decline in the number of cardiac mitochondria, mitochondrial DNA copy number, and superoxide dismutase 2 protein expression [87].

#### 2.7.3. Epithelial Sodium Channel Blockers

Amiloride is an epithelial sodium channel blocker that acts as a potassium-sparing diuretic and natriuretic and is used in the treatment of hypertension, congestive heart failure and hepatic cirrhosis with ascites as an adjuvant to loop diuretics [285]. In bovine submitochondrial particles and in bacterial membranes, amilorides were reported to inhibit bacterial and mitochondrial NADH-quinone oxidoreductase (complex I) [198]. In clonal untransformed and cancer cells, ethyl isopropyl amiloride was found to elicit a significant inhibition of oxidative phosphorylation together with increased mitochondrial fusion, suggested by an alteration in mitochondrial dynamics that includes an increase in elongated mitochondrial networks [199]. Ethyl isopropyl amiloride did not change the mitochondrial membrane potential [199]. In rat articular chondrocytes amiloride was shown to elicit a protective effect against acid-induced apoptosis by attenuating the mitochondrial membrane potential dissipation, by regulating the Bcl-2 family gene mRNA expression and the activity of caspase 3/9 [200].

### 2.8. Statins

Statins are hydroxymethylglutaryl-coenzyme A reductase inhibitors used as the first-line treatment in modulating cholesterol levels in cardiac and metabolic diseases [286]. They act by lowering the liver synthesis and by enhancing the plasma clearance of LDL-cholesterol [286]. Despite all beneficial effects, statins may cause adverse effects. Mitochondrial dysfunction emerged as a major pathomechanism underlying statin toxicity due to coenzyme Q10 level reduction, inhibition of respiratory chain complexes, membrane depolarization, induction of mitochondrial apoptosis, dysregulation of calcium metabolism, and fatty acid oxidation [201,202,203,204].

The mitochondrial toxicity induced by statins was first shown in rat myoblasts and isolated rat skeletal muscle mitochondria where exposure to lipophilic statins (cerivastatin, fluvastatin, atorvastatin, simvastatin) elicited inhibition of ETS complexes I, III, IV, uncoupling of oxidative phosphorylation (cerivastatin), decreased mitochondrial β-oxidation, dissipation of the mitochondrial membrane potential together with an increase in mitochondrial swelling, cytochrome c release, DNA fragmentation and apoptosis. On the contrary, hydrophilic pravastatin was significantly less toxic [205]. In isolated endothelial mitochondria, atorvastatin, but not pravastatin, impaired oxidative phosphorylation at the level of the respiratory chain, mostly at complex I and complex III and at the level of ATP synthesis [206]. Moreover, atorvastatin caused mitochondrial damage by reducing the mitochondrial membrane potential, enhancing the ROS generation, inducing loss of outer mitochondrial membrane integrity and thereby cytochrome c release as well as by disturbing Ca^2+^ mitochondrial homeostasis [206]. The same group also reported that chronic exposure to atorvastatin at physiological concentrations (100 nM) reduced maximal respiration (due to supercomplexes rearrangement) and the cellular coenzyme Q10 content in endothelial cells [287]. Mitochondrial dysfunction induced by statins (atorvastatin, simvastatin, and lovastatin) was also previously reported in the literature in rat hepatocytes via increased ROS generation, lipid peroxidation and mitochondrial depolarization [288]. Similar results were found in atorvastatin-treated pancreas mitochondria [289]. Recently, in human platelets, atorvastatin, simvastatin and cerivastatin were also reported to induce a significant reduction in OXPHOS coupling efficiency (a measure of ATP generating respiration) by inhibiting the electron transport, mainly through the reduction in NADH-linked respiration and by increasing uncoupling (except for simvastatin). Additionally, simvastatin also elicited the inhibition of succinate-linked respiration [290]. Again, the latter in vitro study was a drug toxicity study, recapitulating a condition that might occur when statins accumulate due to impaired metabolism, which explains the higher doses tested as compared to their therapeutic plasma range (1–15 nmol/L) [291].

In patients treated with therapeutic doses of either atorvastatin or rosuvastatin, intact human platelet mitochondrial respiration was not significantly affected [292,293], as opposed to permeabilized platelet respiration, where decreased complex I-linked respiration was noticed [292]. Gvozdjakova et al. found that atorvastatin and fluvastatin treatment caused positive effects on platelet mitochondrial respiratory chain Complex I-linked respiration and ATP production in patients with different pathologies (e.g., diabetes, nephropathy, or dialysis), suggesting that in vivo effects of statins on NADH-linked respiration might be compensated [294]. Recently, it has been reported that chronic treatment with simvastatin at therapeutic concentrations enhanced mitochondrial respiration and complex I and IV activity in peripheral blood mononuclear cells and platelets, but it also increased the production of mitochondrial superoxide as an adverse effect [207]. On the contrary, in a previous study [295] simvastatin therapy was found to impair complex II-linked respiration.

### 2.9. Direct Vasodilators

#### 2.9.1. Organic Nitrates

Organic nitrates (nitroglycerine, isosorbide-5-mononitrate, isosorbide dinitrate, pentaerythrityl tetranitrate) are potent vasodilators that are used successfully in patients with heart failure, coronary artery disease and hypertension [143]. Their effect is mediated by nitric oxide release in response to intracellular bioactivation (the mitochondrial aldehyde dehydrogenase [ALDH-2] for nitroglycerin and pentaerythrityl tetranitrate), activation of guanylyl cyclase enzyme, reduction in intracellular calcium, resulting in vascular smooth muscle relaxation [143]. Long-term administration of organic nitrates is associated with development of tolerance and endothelial dysfunction, which is linked to increased intracellular reactive oxygen production [143]. Sources of reactive oxygen species include mitochondria, NADPH oxidases, and nitric oxide synthase [143]. Different mechanisms are involved in nitroglycerin mitochondrial ROS production: premature release of partially reduced oxygen from mitochondrial complex I or III, lipid peroxidation, decreasing in mitochondrial membrane potential, mitochondrial swelling [144]. Oxidative stress may decrease nitroglycerine bioactivation by inhibiting ALDH-2 or by reducing essential repair cofactors such as lipoic acid [296]. Because isosorbide-5-mononitrate and isosorbide dinitrate do not undergo mitochondrial metabolism, these findings apply only in nitroglycerine tolerance [297]. Plasma concentrations of nitrates cannot be correlated with their effects, due to the tolerance that develops rather fast after drug administration [298].

#### 2.9.2. Molsidomine

Molsidomine, a sydnones drug which has similar properties as organic nitrates was also reported to increase oxidative stress and thereby cause development of tolerance and endothelial dysfunction [117]. Linsidomine (SIN-1), the active metabolite of molsidomine, is a peroxy-nitric donor that is able to release nitric oxygen in the presence of molecular oxygen [299]. In a recent study performed in isolated rat brain mitochondria, SIN-1 lowered the mitochondrial respiratory function, but it did not affect the mitochondrial membrane potential, mitochondrial protein nitrotyrosination or the mitochondrial superoxide levels [145]. In previous studies performed in isolated brain mitochondria from adult male CF-1 mice (64) and in isolated healthy spinal cord mitochondria from young adult female Sprague–Dawley rats (65), exposure to SIN-1 was reported to induce mitochondrial oxidative damage and complex I dysfunction by dose-dependently reducing the respiratory control ratio together with an increase in state II respiration, and a significant decrease in states III and V [146,147]. Additionally, significant increases in mitochondrial 3-nitrotyrosine content were showed [146,147]. In human spermatozoa, SIN-1 was found to decrease the mitochondrial membrane potential and ATP synthesis by inhibiting both glycolysis and OXPHOS [148].

#### 2.9.3. Hydralazine

Hydralazine is a direct arteriole vasodilator used in the management of hypertension and chronic heart failure [300]. Recently it has been demonstrated that in addition to its antioxidant and anti-apoptotic effects [208,209], acute administration of hydralazine inhibited dynamin-related protein 1-mediated mitochondrial fission induced by oxidative stress, preserved mitochondrial fusion events, and decreased cell death in isolated adult murine ventricular cardiomyocytes subjected to ischemia/reperfusion injury [210]. Dehghan et al. demonstrated that hydralazine improved mitochondrial function through a protein kinase A-, Sirtuin 1-, and 5-dependent mechanism to promote longevity in Caenorhabditis elegans using in vitro and in vivo models [211]. In human neuroblastoma SH-SY5Y and mouse myoblast C2C12 cells, hydralazine treatment was found to improve mitochondrial function and to promote mitochondrial biogenesis via increased activity of the ETS complexes, increased ATP production and enhanced mitochondrial membrane potential together with an increase in the mtDNA/nDNA ratio and in the mitochondrial mass [211]. Additionally, the same group reported that when applied in higher doses (above 10 µM) hydralazine inhibited respiration in vitro (revealed by a decreased oxygen consumption rate), but also elevated mitochondrial membrane potential and induced a time-dependent activation of complex IV, thus suggesting that mitochondrial function was not impaired at the same doses [211]. The effects of high concentrations of hydralazine (>200 µM) were also shown in leukemic T cells where hydralazine caused mitochondrial apoptosis by inducing Bak activation and loss of the mitochondrial membrane potential as well as an increased accumulation of ROS [212].

#### 2.9.4. Sodium Nitroprusside

Sodium nitroprusside (SNP) is a commonly used rapid-acting vasodilator agent in the treatment of hypertension emergencies [301] and as a donor of nitric oxide in experimental models [302]. SNP was found to induce severe mitochondrial damage by lowering the mitochondrial membrane potential and by reducing the ATP generation in neuronal PC12 and HepG2 liver cells [149]. In rat chondrocytes, SNP induced mitochondrial apoptosis mediated via reduced mitochondrial membrane potential, downregulated expression of B-cell lymphoma 2 (Bcl-2) level and upregulated expression of Bcl-2-associated X protein (Bax), cytochrome c, caspase-9 and caspase-3 levels [150]. In rat cardiomyocytes, SNP determined mitochondrial alterations, disintegration of sarcomeric alignment and ultimately cell death [302]. SNP severely damaged cardiac H9c2 cells by activating the c-Jun NH2-terminal kinase (JNK) and p38 mitogen-activated protein (MAP) kinase and by decreasing mitochondrial anti-apoptotic proteins (Bcl-2 and Mcl-1 levels) in H9c2 cells [303].

#### 2.9.5. Minoxidil

Minoxidil is a vasodilator that acts by opening the ATP-sensitive potassium channels in vascular smooth muscle cells and is used as an anti-hypertensive agent and to slow or stop hair loss [304]. Minoxidil was recently reported to arrest tumor growth in a xenograft model of ovarian cancer by disrupting the mitochondria and DNA structure and by activating a caspase-3 independent cell death pathway [151]. Minoxidil-induced mitochondrial damage in ovarian cancer cells was revealed by severe mitochondrial morphological abnormalities, and increased electron leak resulting in increased mitochondrial production of superoxide ions [151].

### 2.10. Biguanides

Metformin, the oldest first-line antidiabetic drug, has partially elucidated pleiotropic effects that target mitochondria when experimentally applied in either therapeutic or toxic doses, as recently reviewed by ref. [305]. While the inhibition of complex I of ETS has been the unequivocally demonstrated as therapeutic effect and lactic acidosis as toxic one, recent work has reported the ability of metformin (applied ex vivo in a therapeutic relevant concentration, 10 microM) to decrease the expression of monoamine oxidase (MAO, an enzyme at the outer mitochondrial membrane) and the related oxidative stress in both ventricular [306] and aortic [307] murine samples.

### 2.11. Sodium-Glucose Cotransporter 2 (SGLT2) Inhibitors

SGLT2 inhibitors have recently emerged as oral anti-diabetic drugs that reduce the risk of cardiovascular events and heart failure hospitalizations irrespective of diabetic state [308,309]. In order to explain the success of these drugs in alleviating the heart failure progression, various mechanisms have been suggested, including increased natriuresis, blood pressure lowering, favorable changes in the renin–angiotensin–aldosterone axis, ameliorated renal function and attenuated oxidative stress, but the exact pathomechanism remains to be fully understood [310,311].

#### 2.11.1. Empagliflozin

Empagliflozin was reported to improve mitochondrial function and biogenesis by increasing state 3 respiratory rate, mitochondrial membrane potential, mitochondrial biogenesis-related protein expression: PGC-1α, NRF-1, Tfam and mitochondrial fusion-fission protein expression: dynamin-related protein 1, mitofusin 1 and optic atrophy-1 in the atria of high-fat diet/streptozotocin-induced diabetic rats, and thereby to prevent atrial structural and electrical remodeling [88]. Also, as demonstrated by Mizuno et al., empagliflozin normalized the size and number of mitochondria in diabetic hearts after a myocardial infarction by suppressing ROS generation and by restoring autophagy [89]. Even in a non-diabetic context, empagliflozin improved cardiac function and ameliorated remodeling in rats with left ventricular dysfunction after myocardial infarction by decreasing mitochondrial DNA damage and oxidative stress, by enhancing mitochondrial biogenesis, mitochondrial respiratory capacity and by restoring cardiac glucose and fatty acid oxidation [90,91,92]. In renal cell models, empagliflozin elicited a protective effect against mitochondrial fragmentation, mediated by repression of dynamin-related protein 1 through AMPK activation [312,313,314]. Recently, using both in vivo and in vitro models, empagliflozine was indicated as a promising anti-obesity treatment being capable of inducing white adipocyte browning together with enhanced mitochondrial biogenesis and fusion and improved mitochondrial function, effects mediated through the AMPK signaling pathway and via PGC-1α [93].

#### 2.11.2. Dapagliflozin

Recently, in rat liver mitochondria, dapagliflozin was reported to elicit an antioxidant effect when applied in a concentration of 10 μM by significantly reducing the rate of H_2_O_2_ generation while higher concentrations to 50 μM resulted in inhibition of mitochondrial respiration in states 3 and 3UDNP and in lowered Ca^2+^ retention capacity of rat liver mitochondria [213]. In addition, in human proximal tubular cells, dapagliflozin protected against oxidative stress-induced cell damage by decreasing cytosolic and mitochondrial ROS production and by altering Ca^2+^ dynamics (enhanced the basal intracellular Ca^2+^ in proximal tubular cells, but did not modify Ca^2+^ release from endoplasmic reticulum and store-operated Ca^2+^ entry) [214]. In a rat model of ischemia/reperfusion injury, acute dapagliflozin administration induced cardioprotective benefits by alleviating mitochondrial function, biogenesis and dynamics, as indicated by a decrease in mitochondrial swelling and ROS generation, an enhanced expression of carnitine palmitoyltransferase I (a cardiac mitochondrial metabolism-related protein involved in cardiac fatty acid oxidation) and mitochondrial complex I of the electron transport chain and by increased expression of optic atrophy 1, a mitochondrial fusion protein [215]. In addition, chronic dapagliflozin treatment for 28 days in metabolic syndrome rats subjected to cardiac ischemia/reperfusion injury attenuated the increase in mitochondrial ROS synthesis, depolarization and swelling. Moreover, dapagliflozine reduced mitochondrial fission and increased mitochondrial fusion, as indicated by decreased DRP1 and increased MFN2 and OPA1 protein expression [216]. Similar results were previously reported in insulin-resistant metabolic syndrome rats without ischemia/reperfusion injury [315]. In a mice model of streptozocin-induced diabetes, Belosludtsev et al. showed that dapagliflozine improved the number and ultrastructure of liver mitochondria, upregulated the expression of PPARGC1a, Mfn2 and Drp1 proteins, decreased lipid peroxidation and normalized the respiratory control ratio and calcium retention capacity, while no effects were reported in the healthy animals [217].

#### 2.11.3. Canagliflozin

In an in vitro human renal proximal tubule epithelial cell model system canagliflozin, but not dapagliflozin or empagliflozin inhibited glutamate dehydrogenase and complex I of the mitochondrial ETS when applied in clinically relevant concentrations [218]. In another study, Hawley et al. found that canagliflozin but not dapagliflozin or empagliflozin activated the AMP-activated protein kinase pathway in vivo via inhibition of Complex I of the respiratory chain resulting in enhancement of cellular AMP or ADP, independent of its effect on glucose uptake [316]. The inhibition of complex I by canagliflozin was also reported in prostate and lung cancer cell lines [317]. On the contrary, in breast cancer cells canagliflozin inhibited oxidative phosphorylation, but at the level of complex II of the respiratory chain and only in the situation of very high flux through the electron transport chain (state 3 and in the presence of FCCP) [219]. In addition, a study performed both in vivo and in vitro found that canagliflozin ameliorated obesity by improving mitochondrial biogenesis, function and fatty acid oxidation in adipose tissue and adipocytes via PPARα [220].

### 2.12. Glucagon-like Peptide-1 Receptor Agonists (GLP-1 RAs)

GLP-1 Ras have emerged as a new antidiabetic drug class with cardiovascular benefits, through other mechanisms than glycemic control, being recommended since 2019 as a first-line therapy for type 2 diabetes patients with known cardiovascular disease or those at high risk [318,319].

#### 2.12.1. Liraglutide

In a HepG2 cell model of non-alcoholic steatohepatitis, liraglutide was shown to decrease lipid accumulation and to alleviate mitochondrial dysfunction, ROS and to enhance mitophagy [94]. In addition, liraglutide elicited a protective effect against chronic hypoxic damage via mitophagy activation through amplified SIRT1/Parkin expression, resulting in reversed cellular ATP production, decreased oxidative stress, balanced redox response, attenuated mitochondrial damage and apoptosis in cardiomyocytes [95]. In pulmonary arterial smooth muscle cells, liraglutide caused inhibition of platelet-derived growth factor BB-induced mitochondrial ROS production, mitochondrial membrane potential imbalance, NOX1 expression, and mitochondrial fission Drp1 and also inhibition of autophagy-related protein (Atg)-5, Atg-7, Beclin-1 and LC3-β, leading to reduced proliferation of these cells [320]. In human renal mesangial cells liraglutide was reported to protect against hyperglycemia-induced cell death by alleviating mitochondrial dysfunction, mitochondrial potential decrease, mPTP opening, increased ROS generation and mitochondrial apoptosis via upregulating Sirt3 expression [96]. Liraglutide treatment also induced cardioprotection in high-carbohydrate induced metabolic syndrome rats by attenuating electrical and intracellular Ca^2+^ abnormalities as well as mitochondrial impairment [97]. In an acute mouse model of Parkinson’s disease, liraglutide decreased apoptosis, normalized mitochondrial dynamics, regulated mitophagy by increasing autophagy flux, and lowered oxidative stress [98].

#### 2.12.2. Exenatide

In H9c2 cardiomyocytes subjected to hypoxia/reoxygenation exenatide reduced mitochondrial abnormalities and oxidative stress, increased ATP production, the activity of mitochondrial ATPase and mitochondrial membrane potential and reduced mitochondrial calcium overload and prevented the opening of mPTP, therefore exerting cardioprotective effects by improving mitochondrial function [99]. Lee KH et al. previously showed in a rat model of ischemia/reperfusion injury that exenatide improved morphological and structural changes of mitochondria [100]. Exenatide was also reported to prevent obesity-induced mitochondrial dysfunction via activating SIRT1-PGC-1α signaling, and therefore ameliorating mitochondrial membrane potential decrease, suppressing mitochondrial ROS production and decreasing cell apoptosis in renal tubular epithelial cells both in vitro and in vivo [321]. Other studies also proved the beneficial effects of exendin-4 and liraglutide in ameliorating oxidative stress in diabetic mice by decreasing the ROS level, while preventing mitochondrial dysfunction [322,323].

#### 2.12.3. Dulaglutide

Dulaglutide was reported to alleviate TNF-α-induced mitochondrial dysfunction and oxidative stress in human fibroblast-like synoviocytes by rescuing mitochondrial membrane potential in a dose-dependent manner, by restoring the ROS levels and by increasing the level of the antioxidant GSH [101]. Additionally, it was recently shown that dulaglutide treatment increased the expression of PGC-1α, a master regulator of mitochondrial biogenesis, and the expression of Opa-1, a mitochondrial fusion protein that stabilizes mitochondrial DNA in aged mice [324].

#### 2.12.4. Semaglutide

In lipopolysaccharides treated H9C2 cardiomyocytes, semaglutide was shown to activate the AMPK pathway, to improve autophagy and decrease ROS [102]. In a mouse model of Parkinson’s disease, semaglutide reduced oxidative damage by reducing lipid peroxidation and inhibited the mitochondrial mitophagy signaling pathway while increasing autophagy [103].

#### 2.12.5. Lixisenatide

In human umbilical vein endothelial cells, lixisenatide promoted mitochondrial biogenesis and function through activating the PGC-1α signaling pathway as indicated by increased ratio of mitochondrial-to-nuclear DNA (mtDNA/nDNA), mitochondrial mass, cytochrome B expression, and citrate synthase activity, as well as by enhanced mitochondrial respiration rate and ATP generation [104]. Moreover, lixisenatide alleviated oxidative stress, rescued mitochondrial membrane potential, and arrested cell death in fibroblast-like synoviocytes [105].

## 3. Conclusions

In this review, we attempted to emphasize the complexity of mitochondrial effects of the therapeutic armamentarium currently used in cardiovascular diseases as promising prospects for future translational research into safety pharmacology and drug development. The need for the assessment of mitochondrial toxicity by means of modern testing platforms should be included in preclinical safety pharmacology in order to prevent drug attrition during development, and also decrease the risk of side-/off target deleterious effects, has been recently emphasized by a comprehensive review [325]. While the concept of ”clinical trial in a dish” for drug development is strongly supported by the pharmaceutical industry [326], it has to be mentioned that it will never be able to appropriately recapitulate the complexity of the clinical situation where both disease and ageing-related neurohormonal activation/impaired signaling occur. Indeed, drug-induced mitochondrial dysfunction is more frequent/severe in the elderly population due to both the age-related decline in mitochondrial function and polypharmacy in the setting of comorbidities [15,327]. Moreover, a rigorous design of pre-clinical studies should take into account the clinical relevant dosage and route of administration and, more important, the sex differences [328], since it has been reported that women experience adverse drug reactions nearly twice as often as men [329]. In addition, since most cardiovascular medications interfere with several pathways that regulate mitochondrial homeostasis, preclinical assessment of the organelle toxicity should consider investigation of putative drug-drug interactions for the most common therapeutic associations in everyday practice.

Last but not least, in the past decades, mitochondria have become the target for innovative therapies including molecules with improved pharmacological features [330] and nanocarriers in cardiovascular pathologies, yet no drugs are available so far in clinical setting [25,331]. These therapeutic strategies should be targeted at supporting different mitochondrial pathways modified by the disease per se and/or by the treatment within cardiomyocytes, while also considering the non-cardiomyocyte cells (e.g., fibroblasts, endothelial cells, platelets, immune/inflammatory cells) that critically contribute to the pathophysiology of the disease/its complications.

There is an unmet need for a sustained, collaborative research effort of academia, industry and health professionals in order to expand our understanding of how drugs affect mitochondrial function and allow the identification of the off-target effects of existing medications. This is crucial not only for patient safety but also for discovering novel indications for the available drugs in line with the concept of drug repurposing.

## Figures and Tables

**Table 1:** Beneficial mitochondrial effects of cardiovascular drugs.

Class of Drugs	Drug Name	Mitochondrial Effects	Experimental Model	References
		**Antiarrhytmics**		
Class II (β-blockers)	Timolol	Prevention of oxidative damage (25–5000 µM) Prevention of lipid peroxidation (5 mg/kg body weight, 9 months)	in vitro in vivo (female rat model of aging-related altered left ventricular function)	[28,29,30,31]
Class III (K-channel blockers)	Ibutilide	Attenuation of oxidative stress (10^−8^ to 10^−3^ mol/L) Inhibition of mitochondrial-related apoptosis Increase in glutathione peroxidase and superoxide dismutase levels (10^−8^ to 10^−3^ mol/L)	in vitro (rat cardiomyocytes)	[32]
	Sotalol	No mitochondrial dysfunction (15–240 µM)	in vitro (human platelets)	[33]
	Dofetilide	Correction of the calcium handling (2 mg/kg, 3 days; 10^−6^–10^−8^ mol/L) Correction of NADPH oxidase (2 mg/kg, 3 days; 10^−6^–10^−8^ mol/L)	in vivo (heart failure rat model) in vitro (primary neonatal cardiomyocytes)	[34]
Class IV (Ca-channel blockers)	Verapamil	Inhibition of lipid peroxidation (7 mg/kg) Antioxidant enzyme activity (10 mg/kg twice) Reduction in apoptosis (10 mg/kg twice) Reduction in ROS formation and cytochrome c release (10 mg/kg twice) Increase the ATP concentration (10 mg/kg twice) Reduction in mitochondrial swelling (10 mg/kg twice) Inhibition of mitochondrial membrane potential decrease (10 mg/kg twice)	in vivo (streptozotocin-induced diabetic rats) in vivo (rat model of forebrain ischemia/reperfusion)	[35,36]
	Diltiazem	Protection of mitochondrial integrity (0.1–0.5 µmol/L) Conservation of high-energy phosphate levels (200 µg/kg bolus + 15 µg/kg/min continuous iv infusion) Prevention of mitochondrial swelling (7.5 µM) Prevention of mitochondrial Ca^2+^ increase (7.5 µM) Reduction in lipid peroxidation (5 × 10^−7^M) Decrease apoptosis (10 µmol/L)	ex vivo (drug administered during reperfusion in a rabbit model of heart ischemia/reperfusion) in vivo (rabbit model of myocardium ischemia/reperfusion) ex vivo (drug added before ischemia in a rat model of heart ischemia/reperfusion) ex vivo (reperfused isolated rabbit hearts) in vitro (rat hepatocytes)	[37,38,39,40,41]
Angiotensin-converting enzyme inhibitors (ACEI)	Zofenopril	Prevention of mitochondrial calcium overload (10^−9^–10^−4^ M) Maintenance of oxidative phosphorylation (10^−9^–10^−4^ M) Maintenance of ATP production (10^−9^–10^−4^ M) Preservation of membrane integrity (10^−9^–10^−4^ M) Decrease oxidative stress (10^−9^–10^−4^ M)	ex vivo (rabbit model of myocardium ischemia/reperfusion)	[42]
	Perindopril	Decreased ROS synthesis (2 mg/kg/day, 6 weeks) Increased antioxidant enzymes (2 mg/kg/day, 6 weeks) Increased number of mitochondria (2 mg/kg/day, 6 weeks) Alleviation of mitochondrial ETS dysfunction (2 mg/kg/day, 6 weeks) Increase ATP production (2 mg/kg/day, 6 weeks) Reduction in apoptosis (2 mg/kg/day, 6 weeks) Increased calcium retention capacity (0.2 mg/kg)	in vivo (rat model of isoprotenerol-induced cardiomyopathy) in vivo (drug administered prior to ischemia in a pig model of heart ischemia/reperfusion)	[43,44]
	Trandolapril	Increase in ETS complexes I, II and IV activities (4–6 mg/kg/day, 12 days) Attenuation of oxidative stress (4–6 mg/kg/day, 12 days) Reduction in lipid peroxidation (4–6 mg/kg/day, 12 days) Improvement of mitochondrial oxygen consumption rates (3 mg/kg/day, 6 weeks) Increase in ATP production (3 mg/kg/day, 6 weeks)	in vivo (in a rat model of 3-nitropropionic acid induced brain lesions) in vivo (rat model of failing heart following acute myocardial infarction)	[45,46,47]
	Enalapril	Enhance antioxidant defenses (20 mg/L in drinking water, 11 weeks) Decreased ROS production (10 mg/kg/day, 14 days) Increased mitochondrial mass/biogenesis (20 mg/kg/day, 3 months) Promotion of mitochondrial fusion and autophagy (20 mg/kg/day, 3 months) Reduction in lipid peroxidation (10 mg/kg/day, 12 weeks) Improvement of mitochondrial respiratory efficiency (10 mg/kg/day, 10 weeks)	in vivo (mouse tissues) in vivo (rat kidney mitochondria) in vivo (aged rat hearts) in vivo (rat model of heart failure) in vivo (rat model of doxorubicin-induced cardiomyopathy)	[48,49,50,51,52]
Angiotensin receptor blockers (ARBs)	Valsartan	Improvement of mitochondrial biogenesis and mitophagy (320 mg/day, 4 weeks) Increase mitochondrial respiration (15 mg/kg/day, 4 months) Reduction in mitochondrial oxidative stress (30 mg/kg/day in drinking water, 3 weeks) Increase in mitochondrial β-oxidation (30 mg/kg/day in drinking water, 3 weeks)	in vivo (pig model with renovascular hypertension) in vivo (rats with type 2 diabetes) in vivo (rats with elevated levels of angiotensin II)	[53,54,55]
	Losartan	Reduction in oxidative stress (40 mg/kg/day, 6 months) Increased mitochondrial membrane potential (40 mg/kg/day, 6 months) Amelioration of mtDNA content decrease (30 mg/kg/day, 16.5 months) Improvement of mitochondrial biogenesis (100 mg/L in drinking water, 30 days)	in vivo (spontaneously hypertensive rats) in vivo (aged rats) in vivo (obese mice)	[56,57,58]
	Candesartan	Decreased ROS production (10 μmol/L) Regulation of mitochondrial dynamics (10 μmol/L) Improvement of mitochondrial structure and dynamics (2 mg/kg/day, 8 weeks) Increased mitochondrial membrane potential (2 mg/kg/day, 8 weeks) Alleviation of mitochondrial ETS dysfunction (Complex I, II, III, and IV) (0.1–0.3 mg/kg, 7 days)	in vitro (vascular smooth muscle cells) in vivo (spontaneously hypertensive rats) in vivo (rat model of cerebral ischemia)	[59,60,61]
	Irbesartan	Inhibition of mitochondrial apoptosis (50 mg/kg/day) Increase ATP production (10 nM) Increased mitochondrial membrane potential (10 nM) Decreased ROS production (10 nM)	in vivo (rat model of sleep apnea) in vitro (human and mouse model of non-alcoholic fatty liver disease)	[62,63]
	Telmisartan	Upregulation of mitochondria-specific genes expression (3–10 mg/kg) Increased mitochondrial membrane potential Decreased oxidative stress (5 mg/kg/day, 12 weeks) Modulation of mitochondrial Ca^2+^ homeostasis (5 mg/kg/day, 12 weeks) Enhancement of ATP synthesis (1–10 µM) Increase in mitochondrial complex II activity (1–10 µM) Reduction in apoptosis (1–10 µM)	in vivo (mouse model of Parkinsonism) in vitro (renal glomerular endothelial cells exposed to high glucose) in vivo (hypertensive rats) in vitro (human vascular smooth muscle cells)	[64,65,66,67]
	Olmesartan	Increase in mitochondrial ETS activities (complex I, II) (10 mg/kg/day, 6 weeks) Reduction in oxidative damage (3 mg/kg/day in drinking water, 8 weeks) Improvement of ADP-dependent mitochondrial respiration (3 mg/kg/day in drinking water, 8 weeks)	in vivo (obese insulin resistant rats exposed to an acute glucose load) in vivo (mice model of high-fat diet-induced diabetes)	[68,69]
	Azilsartan	Decreased ROS production (0.1–10 µM) Inhibition of lipid peroxidation (0.1–10 µM) Increased mitochondrial membrane potential (0.1–10 µM) Preservation of ATP production (0.1–10 µM) Reduction in mitochondrial swelling (0.1–10 µM) Alleviation of ETS complexes I, II and IV dysfunction Increased mitochondrial respiration (2–4 mg/kg) Inhibition of apoptosis (2–4 mg/kg) Increased glutathione level (2–4 mg/kg)	in vitro (murine brain endothelial cells) in vivo (rat model of cerebral ischemia)	[70,71]
Angiotensin receptor neprilysin inhibitor (ARNi)	Sacubitril/Valsartan	Attenuation of oxidative stress (68 mg/kg/day, 10 weeks) Improvement of mitochondrial state-3 respiration (100 mg/day, 3 months) Increased mitochondrial membrane potential (100 mg/day, 3 months) Prevention of mitochondrial permeability transition pore opening (100 mg/day, 3 months) Increased ATP production (100 mg/day, 3 months) Normalization of complex-I and IV activities (100 mg/day, 3 months) Inhibition of apoptosis (100 mg/day, 3 months)	in vivo (rat model of pressure overloaded hearts) in vivo (dogs with experimental cardiorenal syndrome)	[72,73]
Calcium channel blockers-dihydropyridines	Amlodipine	Increased oxygen consumption in state 3 (0.4 mg/kg) Increased calcium retention capacity (0.4 mg/kg) Reduction in ROS production (0.4 mg/kg) Decrease in mitochondrial swelling (0.4 mg/kg) Antioxidant properties (5 mg/kg/day, 8 weeks) Increased glutathione peroxidase, catalase and superoxide dismutase activity (1 mg/kg, 7 days) Reduction in lipid peroxidation (1 mg/kg, 7 days) Inhibition of apoptosis (1 mg/kg, 7 days) Enhancement of mitochondrial biogenesis (0.1–1000 μM)	ex vivo (pig ischemia/reperfusion model) in vivo (cholesterol-induced rabbit model of atherosclerosis and a liver and a heart rat model of ischemia/reperfusion injury) in vitro (neural stem cells exposed to oxygen glucose deprivation)	[44,74,75,76,77,78]
Antithrombotic agents	Ticagrelor	Increased mitochondrial membrane potential (1 µM) Decreased ROS production (1 µM) Preservation of ATP synthesis (1 µM) Restoration of mitochondria ultrastructural changes (swelling and loss of crista) (1 µM)	in vitro (insulin-resistant H9 c2 cells)	[79,80]
		**Oral anticoagulants**		
Direct oral anticoagulants	Apixaban	Antioxidant properties (60 ng/mL) Reduction in ROS production (60 ng/mL)	in vitro (model of endothelial dysfunction in uremia)	[81]
	Edoxaban	Increase mitochondrial oxygen consumption (1 μmol/L) Improve mitochondrial ATP generation consumption (1 μmol/L)	in vitro (human alveolar epithelial cells)	[82]
		**Diuretics**		
Loop diuretics	Bumetanide	Attenuation of mitochondrial Ca2+ overload (5 μM) Attenuation of mitochondrial membrane potential dissipation (5 μM) Decreased cytochrome c release (5 μM)	in vitro (astrocytes following ischemia)	[83,84]
Antagonists of aldosterone	Spironolactone	Improvement of mitochondrial membrane potential (0.01–1 µM) Increase in ATP synthesis (0.01–1 µM) Inhibition of ROS production (0.01–1 µM) Inhibition of apoptosis (1–10 µM)	in vitro (methylglyoxal exposed osteoblastic cells)	[85,86]
	Eplerenone	Increased number of cardiac mitochondria (100 mg/kg/day, 6 weeks) Increase in mitochondrial DNA copy number (100 mg/kg/day, 6 weeks)	in vivo (aldosterone-infused mice)	[87]
Sodium-glucose cotransporter 2 (SGLT2) inhibitors	Empagliflozin	Improvement of mitochondrial biogenesis (10–30 mg/kg/day, 8 weeks) Increased state 3 respiratory rate (10–30 mg/kg/day, 8 weeks) Increased mitochondrial membrane potential (10–30 mg/kg/day, 8 weeks) Suppression of ROS generation (10 mg/kg/day, 2 weeks) Reduction in mitochondrial DNA damage (30 mg/kg/day, 10 weeks) Reduction in oxidative stress (30 mg/kg/day, 10 weeks) Restoration of fatty acid oxidation (30 mg/kg/day, 10 weeks) Enhancement of mitochondrial fusion (3.8 mg/kg/day, 8 weeks)	in vivo (rat model of high-fat diet/streptozocin-induced diabetes) in vivo (rat diabetic hearts after myocardial infarction) in vivo (rats with left ventricular dysfunction after myocardial infarction)	[88,89,90,91,92,93]
Glucagon-like peptide-1 receptor agonists (GLP-1 RAs)	Liraglutide	Decreased ROS production (50–500 nM) Increased mitophagy (50–500 nM) Alleviation of mitochondrial membrane potential decrease (1–20 nM) Inhibition of mitochondrial permeability transition pore opening (1–20 nM) Inhibition of apoptosis (1–20 nM) Attenuation of Ca^2+^ abnormalities (0.3 mg/kg, 4 weeks) Normalization of mitochondrial dynamics (0.15 mg/kg)	in vitro (HepG2 cell model of non-alcoholic steatohepatitis) in vitro (human renal mesangial cells exposed to hyperglycemia) in vivo (rat model of high-carbohydrate induced metabolic syndrome) in vivo (acute mouse model of Parkinson’s disease)	[94,95,96,97,98]
	Exenatide	Decreased oxidative stress (0.05–0.6 μM) Increased ATP production (0.05–0.6 μM) Increased mitochondrial ATPase activity (0.05–0.6 μM) Increased mitochondrial membrane potential (0.05–0.6 μM) Decreased mitochondrial calcium overload (0.05–0.6 μM) Inhibition of mitochondrial permeability transition pore opening (0.05–0.6 μM) Improvement of morphological and structural changes of mitochondria (10 mg/kg or 0.3 nM)	in vitro (H9c2 cardiomyocytes subjected to hypoxia/reoxygenation) in vivo (rat model of ischemia/reperfusion injury) and ex vivo (Langendorff model)	[99,100]
	Dulaglutide	Increased mitochondrial membrane potential (50–100 nM) Decreased ROS generation (50–100 nM) Increased glutathione level (50–100 nM)	in vitro (human fibroblast-like synoviocytes exposed to TNF-α)	[101]
	Semaglutide	Decreased ROS production (1–5 mmol/L) Improvement of autophagy (1–5 mmol/L) Decreased lipid peroxidation (25 nmol/kg, 30 days)	in vitro (lipopolysaccharides treated H9c2 cardiomyocytes) in vivo (aged mice)	[102,103]
	Lixisenatide	Promotion of mitochondrial biogenesis (5–20 nM) Increased mitochondrial respiration (5–20 nM) Enhancement of ATP generation (5–20 nM) Inhibition of oxidative stress (10–20 nM) Increased mitochondrial membrane potential (10–20 nM)	in vitro (human umbilical vein endothelial cells) in vitro (human rheumatoid arthritis fibroblast-like synoviocytes)	[104,105]

**Table 2:** Deleterious mitochondrial effects of cardiovascular drugs.

Class of Drugs	Drug Name	Mitochondrial Effects/Dosage	Experimental Model	References
β1 and 2 receptor agonists	Isoprotenerol	Inhibition of respiration (85 mg/kg of body weight) Inhibition of ETS complexes I, II, IV and ATP synthase (85 mg/kg of body weight) Stimulation of the mPTP opening (100 mg/kg body weight) Increase in lipid peroxidation (100 mg/kg body weight) Alteration of glutathione status (100 mg/kg body weight) Induction of antioxidant depletion (≅30 μM) DNA damage and apoptotic signaling (≅30 μM) Induction of oxidative stress (30 mg/kg/day, 8 days) Uncoupling of oxidative phosphorylation (1 mg/kg, 10 days) Decrease ATP levels (1 mg/kg, 10 days) Increased expression of inflammatory markers	in vivo (rats) in vivo (rats) in vitro (cardiomyoblasts) in vivo (mice) in vivo (rat model of experimental chronic heart failure)	[106,107,108,109,110,111,112,113,114,115]
	**Antiarrhytmics**			
Class I (Na-channel blockers)	Quinidine	Uncoupling of oxidative phosphorylation (50 mg/kg/day, 5 days/week for 4 weeks) Reduction in mitochondrial creatine phosphate kinase activity (50 mg/kg/day, 5 days/week for 4 weeks) Decrease ATP production (50 mg/kg/day, 5 days/week for 4 weeks) Inhibition of the protein synthesis in heart mitochondria (50 mg/kg/day, 5 days/week for 4 weeks) Inhibition of respiration (1–4 mM in vitro; 75 mg/kg twice a day for 4 days in vivo)	in vivo (rats) in vitro (isolated kidney cortex mitochondria); in vivo (male rats)	[116,117]
	Propafenone	Reduction in mitochondrial membrane potential (10–20 µM) Decrease the expression of apoptotic inhibitors Bcl-xL and Bcl-2 (10–20 µM)	in vitro (esophageal squamous cell carcinoma)	[118]
Class II (β-blockers)	Propanolol	Induction of mitochondrial swelling and cytochrome c release (2.5–20 µg/mL) Activation of caspase cascade and apoptotic cell death (2.5–20 µg/mL) Inhibition of the ETS complex II (2.5–20 µg/mL) Increased ROS formation (2.5–20 µg/mL) Decreased mitochondrial membrane potential (2.5–20 µg/mL) Depletion of the ATP level (2.5–20 µg/mL)	in vitro (rat cardiomyocytes)	[119,120,121,122,123,124]
Class III (K-channel blockers)	Amiodarone	Inhibition of respiration (20–400 µM) Uncoupling of oxidative phosphorylation (20–400 µM) Inhibition of the mitochondrial complexes I and II (20–400 µM) Inhibition of fatty acid ß-oxidation (20–400 µM) Depletion of ATP content (20–400 µM)	in vitro (isolated rat liver mitochondria, human hepatocytes, rat cardiomyocytes, human platelets, peripheral blood mononuclear cells, HepG2 cells)	[125,126,127,128,129,130,131]
	Dronedarone	Inhibition of fatty acid β-oxidation (1–50 µM) Dissipation of the mitochondrial membrane potential Inhibition of respiration (1–50 µM) Inhibition of mitochondrial complex I (1–50 µM) Uncoupling of oxidative phosphorylation (1–50 µM) Decrease in the intracellular ATP content (1–50 µM)	in vitro (rat liver mitochondria, primary human hepatocytes, HepG2 cells, rat cardiomyocytes)	[129,130,131,132]
Antithrombotic agents	Acetyl-salicylic acid	Inhibition of respiration (2–10 mM) Inhibition of ATP synthesis (2–10 mM) Uncoupling of oxidative phosphorylation (2–10 mM) Inhibition of the respiratory chain ATPase Opening of the mitochondrial transition pore (400 µM) Reduction in mitochondrial membrane potential (400 µM) Increase Ca^2+^ release from the mitochondrion (400 µM)	in vitro (isolated rat cardiac mitochondria) in vitro (rat liver mitochondria) in vitro (rat kidney mitochondria)	[133,134,135]
	Clopidogrel	Inhibition of mitochondrial respiratory state 3 and state 4 respiration—in high doses (10 µg/mL) Reduction in glutathione content (10–100 μM) Decreased mitochondrial membrane potential (10–100 μM) Increased ROS production (10–100 μM) Induction of apoptosis (10–100 μM)	in vitro (isolated mice liver mitochondria) in vitro (primary human hepatocytes and HepG2 cells)	[136,137,138]
	Prasugrel and Ticlopidine	Decreased mitochondrial membrane potential (10–100 μM) Increased ROS production (10–100 μM) Induction of apoptosis (10–100 μM)	in vitro (human neutrophil granulocytes and lymphocytes)	[138]
	**Oral anticoagulants**			
Coumarin derivatives	Warfarin	Reduction in ATP content (0.5–1 mM)	in vitro (isolated rat hepatocytes)	[139]
Direct Oral Anticoagulants	Dabigatran	Increased ROS generation (1–100 µM) Decreased mitochondrial membrane potential (1–100 µM) Increased lipid peroxidation (1–100 µM)	in vitro (rat gastric epithelial cell line)	[140]
	**Diuretics**			
Loop diuretics	Furosemide	Inhibition of ETS complex II Inhibition of state 3 (ADP-dependent) respiration (2 × 10^−3^ mol/L)	in vitro (rat kidney mitochondria, rat liver mitochondria)	[141,142]
Direct vasodilators	Organic nitrates	Increase in ROS production (50–5000 μM) Induction of lipid peroxidation (50–5000 μM) Decreased mitochondrial membrane potential (50–5000 μM) Induction of mitochondrial swelling (50–5000 μM)	in vitro (rat heart mitochondria)	[143,144]
	Molsidomine/ Lisindomine	Induction of oxidative stress (1–20 µM) Inhibition of mitochondrial respiration (50 µmol/L) Inhibition of complex I (1–20 µM) Decreased mitochondrial membrane potential (0.2–0.8 mmol/L) Decrease in ATP synthesis (0.2–0.8 mmol/L)	in vitro (isolated rat brain mitochondria) in vitro (human spermatozoa)	[145,146,147,148]
	Sodium nitroprusside	Decreased mitochondrial membrane potential (0.5–5 mM) Inhibition of ATP generation (0.5–5 mM) Induction of apoptosis (1 mM)	in vitro (neuronal PC12 cells and HepG2 liver cells) in vitro (rat chondrocytes)	[149,150]
	Minoxidil	Induction of mitochondrial morphological abnormalities (50 µg/mL) Increased ROS production (50 µg/mL)	in vitro (ovarian cancer cells)	[151]

**Table 3:** Mixed mitochondrial effects of cardiovascular drugs.

Class of Drugs	Drug Name	Beneficial Effects	Experimental Model	Deleterious Effects	Experimental Model	References
	**Antiarrhytmics**					
	Lidocaine	Alleviation of isoflurane-induced mitochondrial structure damage and the decline in mitochondrial membrane potential 40–100 μg/mL) Reversal of isoflurane-induced mitochondrial ETS dysfunction (40–100 μg/mL) Inhibition of isoflurane-induced apoptosis (40–100 μg/mL)	in vitro (H4 cells exposed to isoflurane)	Suppression of the mitochondrial ETS (0.1–10 mM) Decreased mitochondrial membrane potential (0.1–10 mM) Increased ROS production (0.1–10 mM) Inhibition of ATP synthesis (0.1–10 mM) Induction of mitochondrial structural changes and apoptosis (4–4000 µM)	in vitro (neuronal SH-SY5Y cells) in vitro (human neutrophils)	[152,153,154]
	Phenytoin	Decreased cerebral malondialdehyde as marker of oxidative stress Decreased monoamine oxidase A + B activity in an animal model of epilepsy	in vivo	Increased oxidative stress (200–600 µM) Depletion of glutathione (200–600 µM) Increased lipid peroxidation (200–600 µM) Inhibition of respiration (0.025–1 mM) Decreased ATP synthesis (0.025–1 mM) Decreased mitochondrial membrane potential (0.025–1 mM)	in vitro (rat hepatocytes) in vitro (murine hepatic microsomal system)	[155,156,157]
Class II (β-blockers)	Carvedilol	Antioxidant effects (10 µM) Inhibition of lipid peroxidation (1–50 µM) Mild uncoupling of mitochondrial oxidative phosphorylation (10–100 µM) Decrease in ROS production (10–20 µM) Prevention of calcium overload (10–20 µM) Inhibition of NADH dehydrogenase and prevention of oxidative damage (10–20 µM) Inhibition of mPTP (5–20 µM)	in vitro (swine ventricular membranes, rat brain homogenates, human LDL, bovine and human endothelial cells, rat heart mitochondria)	Induction of severe mitochondria damage—mitochondrial swelling, crista damage and formation of myelin figures inside the mitochondria (10 µM)	in vitro (rat C6 glioma cells)	[158,159,160,161]
	Nebivolol	Antioxidant activity (1–2 mg/kg, 8 weeks) Inhibition of NADPH oxidase activity (1–2 mg/kg, 8 weeks)	in vivo (streptozocin treated diabetic rats)	Inhibition of complex I and V (1 µM) Inhibition of respiration (1 µM) Depletion of ATP levels (10 µM) Induction of mitochondrial morphology changes (10 µM) Increased ROS production (10 µM)	in vitro (breast, colon and oral squamous carcinoma cells)	[162,163,164]
	Metoprolol	Increased mitochondrial respiratory control ratio (1 mg/kg -bolus infusion)	in vivo (rat model of ischemia/reperfusion injury)	No protective effect against adriamycin-induced mitochondrial DNA impairment (3 mg/kg/12 h, 12 days)	in vivo (rat model of adriamycin-induced cardiotoxicity)	[165,166,167,168]
	Atenolol	Decrease in membrane fatty acid unsaturation degree of mitochondria (0.1 g/L of atenolol drinking water solution) Reduction in mitochondrial protein oxidative, glycoxidative, and lipoxidative modification (0.1 g/L in drinking water) Reduction in oxidative damage in heart mitochondrial DNA (0.1 g/L in drinking water)	in vivo (rats)	Increased ROS production (2.5–20 μg/mL) Decrease in mitochondrial succinate dehydrogenase activity (2.5–20 μg/mL) Decreased mitochondrial membrane potential (2.5–20 μg/mL) Induction of mitochondrial swelling (2.5–20 μg/mL) Decreased ATP content (2.5–20 μg/mL)	in vitro (isolated rat heart mitochondria)	[119,169,170,171]
	Esmolol	Improvement of mitochondrial morphology (300 μg/kg/min, 48 h) Prevention of apoptosis by decreasing the Bax/Bcl-2 levels (1.75–3.5 mg/Kg/h)	in vivo (spontaneously hypertensive rats) in vivo (early sepsis rats with abdominal infection)	Increased ROS level (5–250 μM) Decreased mitochondrial membrane potential (5–250 μM)	in vitro (human lung fibroblast cells)	[172,173,174,175]
Others	Adenosine	Attenuation of the decline of complex I and mitochondrial NO synthase activities (0.03 μg/kg/min, 65 min) Reduction in mitochondrial phospholipid oxidation (0.03 μg/kg/min, 65 min)	ex vivo (experimental model of rabbit heart ischemia/reperfusion)	Induction of apoptosis (0.1–10 mM) Increased ROS production (0.1–10 mM) Reduction in Bcl-X(L) expression (3 mM) Disruption of mitochondrial membrane potential (3 mM)	in vitro (liver cancer cells) in vitro (HepG2 cells)	[176,177,178,179]
	Digitalis	Enhancement of the efficiency of mitochondrial electron transport and ATP synthesis (1–100 nM in vitro; 1 mg/kg, 5–8 days in vivo)	in vitro (rat cardiomyocytes) in vivo (mice)	Reduction in mitochondrial Ca2+ accumulation (1 µM) Reduction in the NADH/NAD+ redox potential (1 µM) Increased ROS production (1 µM) Decrease mitochondrial membrane potential (0.025–0.2 µM) Induction of mitochondrial related apoptosis (0.025–0.2 µM) Increase Bax/Bcl-2 proportion (50–200 nM) Depletion of ATP (0.03–100 µM)	in vitro (guinea pig ventricular myocytes) in vitro (human non-small cell lung cancer cells A549) in vitro (breast cancer cells) in vitro (HeLa cell line)	[180,181,182,183,184,185]
Angiotensin-converting enzyme inhibitors (ACEI)	Ramipril	Attenuation of lipid peroxidation (10 mg/kg/day, 28 days in vivo; 10 µM in vitro)	in vivo (rat model of rheumatoid arthritis) and in vitro (rat cardiomyocytes)	Inhibition of cardiac uncoupling protein-2 expression (50 µg/kg/day, 4 weeks)	in vivo (rat model of ischemia/reperfusion)	[186,187]
	Captopril	Attenuation of mitochondrial membrane potential dissipation (10 mg/kg, 7–8 days) Increase ATP production (10 mg/kg, 7–8 days) Restoration of mitochondrial oxygen consumption (5 mg/kg, 12 weeks) Antioxidant effect (0.08 mM)	in vivo (rat model of adriamycin toxicity) in vivo (rabbits with experimentally induces hypercholesterolemia) in vitro (rat liver mitochondria)	Decrease in respiration rates (5 mg/kg, 12 weeks) Inhibition of ATP synthase activity (0.1–0.5 mmol/L)	in vivo (rabbits with experimentally induces hypercholesterolemia) in vitro (rat heart mitochondria)	[188,189,190,191,192]
	Lisinopril	Attenuation of oxidative stress (40 mg/L in drinking water) Increase mitochondrial content (40 mg/L in drinking water)	in vivo (rat model of irradiation-induced kidney damage)	Reduction in mitochondrial respiration (50–10,000 ng/mL)	in vitro (Drosophila melanogaster strains)	[193,194]
Direct Oral Anticoagulants	Rivaroxaban	Reduction in ROS generation (300 nM in vitro; 12 mg/kg/day, 28 days in vivo) Antioxidant effects (5.6 mM)	in vitro (advanced glycation end products-exposed proximal tubular cells); in vivo (intermittent hypoxia exposed mice) in vitro (rat kidney mitochondria)	Decrease in mitochondrial succinate dehydrogenase activity (1.4–2.8 mM) Increase ROS production (1.4–2.8 mM) Induction of mitochondrial swelling (1.4–2.8 mM) Reduction in mitochondrial membrane potential (1.4–2.8 mM)	in vitro (rat kidney mitochondria)	[195,196,197]
	**Diuretics**					
Epithelial sodium channel blockers	Amiloride	Attenuation of the mitochondrial membrane potential dissipation (50–200 μM) Inhibition of apoptosis (50–200 μM)	in vitro (rat articular chondrocytes)	Inhibition of mitochondrial NADH-quinone oxidoreductase (complex I) (5–100 µM) Inhibition of oxidative phosphorylation (10 µM) Increased mitochondrial fusion (10 µM)	in vitro (in bovine submitochondrial particles and in bacterial membranes) in vitro (clonal untransformed and cancer cells)	[198,199,200]
Statins		Enhancement of mitochondrial respiration (2.5–10 µM Simva) Increase in complex I and IV activity (2.5–10 µM Simva)	in vitro (peripheral blood mononuclear cells and platelets)	Reduction in coenzyme Q10 level (1–100 µM) Increase in ROS generation (25–700 µM) Inhibition of respiration (1–1000 µmol/L) Inhibition of respiratory chain complexes (1–1000 µmol/L) Uncoupling of oxidative phosphorylation (1–1000 µmol/L) Reduction in ATP production (1–1000 µmol/L) Induction of mitochondrial membrane depolarization (1–1000 µmol/L) Induction of mitochondrial apoptosis (1–1000 µmol/L) Induction of mitochondrial swelling (1–1000 µmol/L) Dysregulation of calcium metabolism (25–700 µM) Induction of fatty acid oxidation (Simva, 80 mg/day, 12 weeks)	in vitro (rat myoblasts, isolated rat skeletal muscle mitochondria, isolated endothelial mitochondria, rat hepatocytes, pancreas mitochondria, human platelets) in vivo	[201,202,203,204,205,206,207]
Direct vasodilators	Hydralazine	Antioxidant properties (10–30 mg/kg) Inhibition of apoptosis (10–30 mg/kg) Inhibition of mitochondrial fission (1 µM) Preservation of mitochondrial fusion (1 µM) Promotion of mitochondrial biogenesis (5–20 µM) Increase in ETS complexes activity (5–20 µM) Increase in ATP production (5–20 µM) Enhancement of mitochondrial membrane potential (5–20 µM) Increase in mtDNA/nDNA ratio (5–20 µM) Increase in mitochondrial mass (5–20 µM)	in vivo (rat model of ischemia/reperfusion) in vitro (isolated murine cardiomyocytes subjected to ischemia/reperfusion injury) in vitro (human neuroblastoma SH-SY5Y and mouse myoblast C2C12 cells)	Inhibition of respiration (10–100 µM) Induction of apoptosis (200–600 µM) Increase ROS production (200–600 µM) Reduction in mitochondrial membrane potential (200–600 µM)	in vitro (human neuroblastoma SH-SY5Y and mouse myoblast C2C12 cells) in vitro (leukemic T cells)	[208,209,210] [211,212]
Sodium-glucose cotransporter 2 (SGLT2) inhibitors	Dapagliflozin	Antioxidant properties (10 µM) Reduction in ROS production (0.1–10 μM) Alteration of Ca^2+^ dynamics (0.01–10 μM) Decrease in mitochondrial swelling (1 mg/kg) Reduction in mitochondrial fission (1 mg/kg/day, 28 days) Increase in mitochondrial fusion (1 mg/kg/day, 28 days) Normalization of respiratory control ratio (1 mg/kg/day, 20 days) Decrease lipid peroxidation (1 mg/kg/day, 20 days)	in vitro (rat liver mitochondria) in vitro (human proximal tubular cells) in vivo (rat model of ischemia/reperfusion injury) in vivo (metabolic syndrome rats subjected to ischemia/reperfusion) in vivo (mice model of streptozocin induced diabetes)	Inhibition of mitochondrial respiration (20–50 µM) Reduction in calcium retention capacity (20–50 µM)	in vitro (rat liver mitochondria)	[213,214,215,216,217]
	Canagliflozin	Improvement of mitochondrial biogenesis (60 mg/kg/day, 14 weeks) Improvement of fatty acid oxidation (60 mg/kg/day, 14 weeks)	in vivo (mice model of high-fat diet induced obesity)	Inhibition of the ETS complex I (10–50 µM) Inhibition of the ETS complex II (50 µM)	in vitro (human renal proximal tubule epithelial cell model system) in vitro (breast cancer cells)	[218,219] [220]

## Data Availability

Not applicable.

## Abbreviations

ACEI	Angiotensin-converting enzyme inhibitors
ADP	Adenosine diphosphate
AMPK	AMP-activated protein kinase
ARBs	Angiotensin receptor blockers
ARNi	Angiotensin receptor neprilysin inhibitor
ATP	Adenosine triphosphate
Bcl-2	B-cell lymphoma 2
Bax	Bcl-2-associated X protein
Cyt. c	Cytochrome c
CI	Complex I
CII	Complex II
ETS	Electron transport system
eNOS-PGC-1α	Endothelial nitric oxide synthase 3-Peroxisome proliferator-activated receptor-gamma coactivator-1alpha
GLP-1 RAs	Glucagon-like peptide-1 receptor agonists
LEAK	Non-phosphorylating respiration
LDL	Low-density lipoprotein
mtDNA	Mitochondrial deoxyribonucleic acid
mPTP	Mitochondrial permeability transition pore
NADH	Nicotinamide adenine dinucleotide reduced form
OXPHOS	Oxidative phosphorylation
RAAS	Renin-angiotensin-aldosterone system
ROS	Reactive oxygen species
SGLT2	Sodium-glucose cotransporter 2 inhibitors
TNFα	Tumor necrosis factor α
